# An ECHO of Cartilage: *In Silico* Prediction of Combinatorial Treatments to Switch Between Transient and Permanent Cartilage Phenotypes With *Ex Vivo* Validation

**DOI:** 10.3389/fbioe.2021.732917

**Published:** 2021-11-15

**Authors:** Sakshi Khurana, Stefano Schivo, Jacqueline R. M. Plass, Nikolas Mersinis, Jetse Scholma, Johan Kerkhofs, Leilei Zhong, Jaco van de Pol, Rom Langerak, Liesbet Geris, Marcel Karperien, Janine N. Post

**Affiliations:** ^1^ Technical Medicine Centre, Department of Developmental BioEngineering, University of Twente, Enschede, Netherlands; ^2^ Department of Formal Methods and Tools, CTIT Institute, University of Twente, Enschede, Netherlands; ^3^ Biomechanics Research Unit, GIGA *In Silico* Medicine, ULiège, Liège, Belgium; ^4^ Dept. of Computer Science, Aarhus University, Aarhus, Denmark; ^5^ Biomechanics Section, Department of Mechanical Engineering, KU Leuven, Leuven, Belgium

**Keywords:** computational model, signal transduction, IGF, BMP7, PTHrP, chondrogenesis, hypertrophy

## Abstract

A fundamental question in cartilage biology is: what determines the switch between permanent cartilage found in the articular joints and transient hypertrophic cartilage that functions as a template for bone? This switch is observed both in a subset of OA patients that develop osteophytes, as well as in cell-based tissue engineering strategies for joint repair. A thorough understanding of the mechanisms regulating cell fate provides opportunities for treatment of cartilage disease and tissue engineering strategies. The objective of this study was to understand the mechanisms that regulate the switch between permanent and transient cartilage using a computational model of chondrocytes, ECHO. To investigate large signaling networks that regulate cell fate decisions, we developed the software tool ANIMO, Analysis of Networks with interactive Modeling. In ANIMO, we generated an activity network integrating 7 signal transduction pathways resulting in a network containing over 50 proteins with 200 interactions. We called this model ECHO, for executable chondrocyte. Previously, we showed that ECHO could be used to characterize mechanisms of cell fate decisions. ECHO was first developed based on a Boolean model of growth plate. Here, we show how the growth plate Boolean model was translated to ANIMO and how we adapted the topology and parameters to generate an articular cartilage model. In ANIMO, many combinations of overactivation/knockout were tested that result in a switch between permanent cartilage (SOX9+) and transient, hypertrophic cartilage (RUNX2+). We used model checking to prioritize combination treatments for wet-lab validation. Three combinatorial treatments were chosen and tested on metatarsals from 1-day old rat pups that were treated for 6 days. We found that a combination of IGF1 with inhibition of ERK1/2 had a positive effect on cartilage formation and growth, whereas activation of DLX5 combined with inhibition of PKA had a negative effect on cartilage formation and growth and resulted in increased cartilage hypertrophy. We show that our model describes cartilage formation, and that model checking can aid in choosing and prioritizing combinatorial treatments that interfere with normal cartilage development. Here we show that combinatorial treatments induce changes in the zonal distribution of cartilage, indication possible switches in cell fate. This indicates that simulations in ECHO aid in describing pathologies in which switches between cell fates are observed, such as OA.

## Introduction

Proper development of cartilage is important for the length of our long bones by anatomical movement of growth plate cartilage and supple joint movement through formation of articular cartilage. Cartilage dysregulation occurs in a variety of diseases, including growth disorders, rheumatic diseases, osteoarthritis, as well as in chondrosarcomas. To understand cartilage disorders and identify new biomarkers or therapies, insight into the dynamics of the cellular networks that control chondrogenesis is necessary.

Cartilage formation is under control of the transcription factor SOX9, that regulates expression of genes important for the cartilage phenotype, including collagen 2 and aggrecan (Goldring, 2012). Mutations in SOX9 are linked to various diseases, including campomelic dysplasia (Foster et al., 1994). Also, Sox9 is sufficient for cartilage formation, since Sox9 misexpression produced ectopic cartilage (Healy et al., 1999). Moreover, loss of SOX9 activity and subsequent decrease in target gene expression is observed in osteoarthritis (Kim et al., 2013) and is correlated to osteoarthritis progression (Zhong et al., 2016a).

In the growth plate, RUNX2 drives proliferative chondrocytes into hypertrophic chondrocytes, which is a prerequisite for bone formation. In these cells, RUNX2 aids bone formation by regulating expression of collagen1, MMP13 and osteocalcin (Ducy et al., 1997). Runx2 is required for bone formation (Otto et al., 1997; Komori, 2010). A tight balance between the activities of these transcription factors is therefore essential for the correct development and maintenance of cartilage and bone tissues. The activities of SOX9 and RUNX2 are regulated by an intricate network of signal transduction pathways, including IHH, PtHrP, FGF, WNT, BMP, TGFbeta, HIF and IGF (Kronenberg, 2003; Zhong et al., 2015). Because of the complexity of the signaling network, it is impossible to predict the effect of network changes (mutations, overexpression, loss of function, etc) on the system as a whole.

Computational models based on systems biology principles may offer general alternatives to time-consuming analytical laboratory experimentation, because the *in silico* execution of a program provides a rapid evaluation of working hypotheses. Effective models have the potential to 1) reduce the costs of expensive *in vitro* and *in vivo* experiments, 2) prevent animal suffering, and 3) allow description of biological processes in humans in which deliberate biological experimentation cannot be performed apart from highly regimented clinical trials. Therefore, computational models have the potential to transform experimental biology by describing and understanding observations and ultimately to predict cell behavior and to assist with the design of new biological experiments. The outcomes of biological experiments will either validate the model or will identify novel mechanisms that can be incorporated in the model, and thus computational modeling enhances the accuracy and predictive potential of biological concepts (Kumar et al., 2006).

Mathematical modeling of the dynamics of biological networks permits formal comparisons of new experimental data to prior knowledge, because formal description of molecular interactions enables assessment of matches between network topology and experimental data. However, often a thorough understanding of specific mathematical languages is required for optimal use of the available tools.

We have previously described the development of a modeling tool referred to as Analysis of Networks with Interactive Modeling (ANIMO) (Schivo et al., 2012, 2014b; Scholma et al., 2014; Schivo et al., 2016). Because ANIMO centers around a visual network representation, it renders intuitive generation and editing of models, and supports the formal exploration of networks by users without a thorough training in mathematical formalisms (Pavelin et al., 2012). For this reason, we have implemented ANIMO as a plug-in to Cytoscape (Shannon et al., 2003), a widely used open-source software platform for static visualization of complex networks. In Cytoscape, the network is drawn as a graph, with nodes and edges representing molecules and interactions respectively. We have previously validated the ANIMO modeling tool using both novel new models and using models that were previously generated using different mathematical formalisms (Schivo et al., 2012, 2014b; Scholma et al., 2014; Schivo et al., 2016).

While cellular networks are infinitely complex, we applied ANIMO to build a dynamic protein activity network for articular chondrocytes referred to as the Executable CHOndrocyte (ECHO) model. Recently, we showed that ECHO predicts the chondrogenic differentiation capacity of multiple relevant human cell types, including pluripotent embryonic and somatic multipotent mesenchymal stem cells (Schivo et al., 2019). Here, we show the building of ECHO from a Boolean model of growth plate cartilage (Kerkhofs et al., 2016). Using model checking (Bartocci et al., 2009; David et al., 2015; Schivo and Langerak, 2017) we prioritized model-predictions that were tested in the wet-lab.

We show that simultaneous inhibition of ERK and activation of the IGF pathway prevented bone formation while enhancing cartilage formation in rat metatarsal explants. In contrast, simultaneous activation of DLX5 and inhibition of IGF via GLI2 prevented cartilage formation and inhibited the growth of metatarsals.

## Methods

### Building Models Using the Tool ANIMO

The modeling tool ANIMO, which stands for Analysis of Networks with Interactive MOdeling, has been developed in a collaborative project between cell biologists and computational scientists. The aim was to create a tool that would provide biologists with the computational support needed to reason on the dynamics of complex cell signaling networks. To make the formal analysis layer implemented in ANIMO available to biologists in a familiar environment, ANIMO is provided as a plug-in to Cytoscape (Shannon et al., 2003), Supplementary Figure S1. The network diagram that can be drawn with Cytoscape’s tools provides the topological information; using ANIMO we add activity information to the network (Schivo et al., 2012, 2014b; Scholma et al., 2014; Schivo et al., 2016). Activity in this case is a very broad term and is used to describe for example active gene expression, but also post-translational modifications such as an active kinase that phosphorylates its target, or a ligand binding to a receptor thereby inducing receptor dimerization and activation.

#### Nodes and Interactions

In the network diagram we have nodes, which represent molecules such as ligands, kinases, mRNA, etc., that are connected via edges, representing the interactions between the nodes. Each node in the network represents the inactive and the active state of the molecule, with the relative amounts of active molecules as percentage of activity represented by the node color. For example: a node with activity level of 30 out of 100 can represent a kinase population, 30% of which is in its “active” state. Nodes can interact through activations or inhibitions, which define how an upstream node influences the activity of the downstream node. This is exemplified as follows: the interaction A → B (read “A activates B”) indicates that node A, if active, will increase the activity level of node B. If we add a second interaction to the example, C ⊣ B (“C inhibits B”), with C also active, then the activity level of B will change depending on the activity levels of A and C, and on their quantitative influence. Node activities range with integer values between 0 and 100, unless otherwise noted, while interactions are described as positive or negative influences.

#### *k*-Parameters

The influence of an interaction is quantified by a parameter *k*, which defines the speed at which that interaction occurs: higher values of *k* give faster interactions. These *k*-values are the only parameters needed in an ANIMO model. The value of the constant *k* can be either given as numeric, or chosen among a pre-defined set of qualitative estimation, choosing from “very slow,” “slow,” “normal,” “fast” and “very fast” (Schivo et al., 2014a). Going back to the example with the interactions A → B and C ⊣ B, suppose we have $k_{\text{A}\to \text{ B}}=0.5$ and $k_{\text{C }⊣\text{ B}}=0.4$ and that both A and C have 100/100 activity. In this case, because $k_{\text{A}\to \text{ B}}>\;k_{\text{C }⊣\text{ B}}$ (i.e., A → B is “faster” than C ⊣ B), the activity level of B will increase over time.

#### Kinetic Scenarios

In ANIMO, kinetic scenarios based on Michaelis-Menten kinetics (Michaelis and Menten, 1913; Michaelis et al., 2011) are defined for each interaction. In biochemical terms, a phosphorylation reaction catalyzed by enzyme E on substrate S can be represented as:

$$E+S+ATP\;⇄ES+ATP\to ES^{P}+ADP\;⇄E+\;S^{P}+ADP$$

The same reaction is abstracted in our model by the corresponding interaction E → S. Each occurrence of the interaction E → S will increase the activity level of S by one discrete step (e.g., from 30 out of 100 to 31 out of 100). The rate R of occurrence of an interaction is defined by the user, who can choose an abstract kinetic scenario from the three available:

1) R = k × [E]: the rate of occurrence depends only on the activity level of the upstream node
2) R = k × [E] × [S]: the rate depends on the activity levels of both participants
3) R = k × [E_1_] × [E_2_]: the rate depends on the activity levels of two user-selected reactants. This scenario can be used to represent the so-called AND gate kinetics, i.e., the case when the activity of a downstream node depends on the simultaneous presence of two upstream nodes. We have shown that the abstraction proposed here preserves ample descriptiveness to capture experimental data in meaningful models (Schivo et al., 2016; Zhong et al., 2017; Schivo et al., 2019).

#### Under ANIMO’s Hood: Timed Automata

All models built with ANIMO are analyzed using the mathematical/formal language of Timed Automata (TA). Technically, the TA models which we automatically produce from an ANIMO model are built to approximate a set of Ordinary Differential Equations (ODEs) in a discrete manner (Schivo and Langerak, 2017). In order to get a complete and precise description of how ANIMO models are translated into TA and how those models approximate ODEs, we refer the interested reader to our previous work (Schivo et al., 2012; Schivo et al., 2014a; Schivo et al., 2014b; Scholma et al., 2014; Schivo and Langerak, 2017), where we show how nodes and interactions are represented, and how TA are used to update the activities along the course of a model simulation.

Node activity levels are represented in the TA model using integer variables and are updated based on the interactions influencing them. These integer variables are each managed by one timed automaton: whenever an automaton reaches its timeout, the corresponding variable is changed by +1 or −1. This means that the corresponding activity level either increases or decreases by 1 at that point in time. The amount of time that needs to pass before a timeout is reached needs to be kept constantly updated and depends on the interactions influencing the node represented by the automaton.

Consider again the example network made of the nodes A, B and C, and the interactions A → B and C ⊣ B. Note that in this network only the value of B can change, because no interaction exists upstream of A or C. This means that from this ANIMO model we will obtain only one timed automaton, which will manage B’s activity level. The aspect of this automaton is very similar to the one represented in Supplementary Figure S1. The most important location of the automaton is labelled “waiting” and is used to wait for the next timeout. This is done by letting time flow and checking the value of clock c. When c exceeds the threshold T, it is possible to leave location waiting and reach a special location (the one with a “C” inside): this allows to update the variable representing B’s activity level [which is done by function reach ()], reset the clock c, and compute the value of the threshold T for the next timeout. The new value of T is computed in function update () taking into account all the interactions influencing B. In this case, the update formula depends on the current values of A’s and C’s activity levels, and on the *k*-parameters $k_{\text{A }\to \text{ B}}$ and $k_{\text{C }⊣\text{ B}}$ . It is in this function that our discrete approximation of the ODEs representing the network (Schivo and Langerak, 2017) is applied. The sign of the next change to B’s activity level (+1 or −1) is also decided by the update () function, taking into account the current conditions. When performing the update we also move back to the waiting location, and send an output signal (denoted by !) on the reaching [1] channel: this allows us to alert any automaton that may depend on B that its activity level has changed. Note that it is also possible to leave the waiting location before clock c has reached its threshold T: this can happen when another automaton has reached its timeout before the current one. This event is detected by waiting for a signal on the reaching [..] channel, using an input action (denoted by ?). In case of such an event, we reach location “responding”: there, an update to the value of T can be made to react to the possibly changed environmental conditions. In our example, location “responding” would be reached in case other interactions had changed A’s or C’s activity levels.

Summarizing, an automaton in our TA model can be involved in two types of events:

- Clock timeout: the value of the managed activity level is updated, and a signal is sent to all interested automata;
- Change in conditions: other automata have changed variables that may influence the value of T, so this needs to be recomputed.

The initial location “start” is used to initialize the threshold T, using the initial activity levels of the nodes.

Note that the behavior described here is *deterministic:* i.e., given a set of *k*-parameters, the analysis of the TA model will always return the same result over any number of simulations. In case non-deterministic behavior needs to be described, it is possible to introduce non-determinism in an ANIMO model. However, we chose not to use this feature when working on ECHO to reduce its complexity.

Here, we provided only an abstract description of how the TA model works, without going into many of the details that make it work. To get a more complete picture, we refer to (Schivo and Langerak, 2017).

#### From Boolean Models to ANIMO Models

As already mentioned, ECHO is based on a previously existing Boolean model (Kerkhofs et al., 2012), which was translated into ANIMO and subsequently refined.

Boolean networks can be translated into ANIMO as follows: Boolean OR gates, such as (A OR B) → C, will be translated into A → C and B → C. This means that whenever either A or B is active, C will be activated, so that reaction effects are always additive. This representation of OR is thus non-exclusive, so C will be activated also if both A and B are active, but in that case the activation will proceed faster. A Boolean AND gate can be explicitly represented with the “AND” approximation described by scenario 3: with (A AND B) → C, C will be activated only if both A and B are active.

It is of importance to note that in ANIMO all nodes that are activated remain active until they are inactivated. To model inactivation events, such as protein/mRNA degradation, receptor internalization, dephosphorylation, etc., inhibitory edges must be added to each node. The parameters for this inhibition depend on the rate of the biochemical reaction that is being represented. For example, dephosphorylation is a fast process, but not faster than phosphorylation as we know that when we quantify protein phosphorylation by for example Western Blot, we identify a peak between 5 and 30 min after cell stimulation that tapers off to zero after one to 2 h.

Combining these basic tools makes the representation of more complex Boolean formulas also possible, even if not always in a straightforward manner. Based on the truth table of the Boolean formula, we made use of “dummy” nodes to define these special behaviors. For example, to represent the inactivation of the “Destruction complex” in the Wnt canonical pathway and the influence that ERK exerts on it, the original model uses the formula: (1-Dsh)*Min ((1.5 - ERK), 1). To obtain the same effect, we defined the subnetwork in Supplementary Figure S2: note the presence of the dummy nodes with fixed value that represent constitutional activity of the Destruction complex.

#### *k*-Parameters in ECHO

Most precise modeling is based on Kd values that are obtained in purified enzyme reactions (for example Kogan et al., 2012). However, for many proteins in our network these values are unknown. In addition, mechanisms such as subcellular localization and competition with other proteins, are not considered when determining the Kd values. What most experimental biologists do know, is the speed of protein phosphorylation observed in western blot experiments, or functional assays. We have described for our cells, that phosphorylation assays measure highest intensities around 15–30 min after stimulation (Scholma et al., 2014; Zhong et al., 2017). Work on protein phosphorylation has been performed since the early 1900s, and enzymatic phosphorylation of proteins has been described since the 1930s (reviewed in Pawson and Scott, 2005). As such, much information exists about the speed of phosphorylation. Textbooks, such as Essential Cell Biology (Alberts et al., 2019) describe protein phosphorylation as a very fast process. Indeed, a report using single cell measurements on a microfluidic chip, has indicated that protein phosphorylation takes place within 30–200 s after cell stimulation for the proteins measured (Blazek et al., 2015), with differences between proteins and even the different phosphorylation sites within the proteins. We therefore chose to use this knowledge and applied it as follows: Protein phosphorylation is represented as a “fast” process in ECHO, thus all phosphorylation reactions, together with reactions with a comparable speed, are represented with a k-value of 1. Those reactions that involve gene transcription are represented as “slow” using a k-value of 0.1. Due to this simplified approach, timing information is not considered in ECHO: all the *in silico* experiments illustrated here are based on letting the model evolve for a time long enough to let it reach an attractor state. The attractor states in ECHO are the same three as in the original model and describe the possible configurations towards which the model can naturally evolve. We named the attractor states based on the activity of the two most important nodes: SOX9+ corresponds to the state where the SOX9 node is active and RUNX2 is not, RUNX2+ denotes RUNX2 activity and SOX9 inactivity, and Null describes a state where all nodes in the network are at 0 activity. Table 1 shows the activity levels of nodes SOX9 and RUNX2 in the three stable states reached in ECHO.

**TABLE 1:** Node activity levels of SOX9 and RUNX2, in the three stable states that can be reached in ECHO.

Stable state name	SOX9 node activity level	RUNX2 node activity level
SOX9^+^	88	0
RUNX2^+^	0	100
Null	0	0

### Model Definition

The description of the model and the simulations is according to the MIASE descriptions (Waltemath et al., 2011). The model validation has been shown before in (Schivo et al., 2019). Model parameters for the AC and GP models have been previously described (Schivo et al., 2019).

A detailed description of the ANIMO modelling approach was published previously (Schivo et al., 2012, 2014b; Scholma et al., 2014; Schivo et al., 2016), and is shortly illustrated in the previous sections.

The base version of ECHO (Growth Plate, GP model) was ported to ANIMO from pre-existing Boolean model and additive models of the growth plate (Kerkhofs et al., 2012; Kerkhofs and Geris, 2015; Kerkhofs et al., 2016). We converted the existing Boolean model into an ANIMO model, which we then named the executable chondrocyte, or ECHO.

The semi-quantitative model on which ECHO is based uses additive functions to represent Boolean-like logic, with node activities in the continuous [0,1] interval. This model is translated into ANIMO’s kinetics by applying the following set of rules:

• All nodes in ECHO have 100 discrete levels of activity. The activity level of a node can be interpreted as concentration on the arbitrary scale from 0 to 100, or as the percentage of active (e.g., phosphorylated) molecules over the whole population, depending on whether a node represents a gene or a protein.
• Two classes of reactions can be identified: slow (e.g., gene expression) and fast (e.g., post-translational modifications). If a reaction can be directly identified as belonging to one of these general categories, the corresponding interaction strength factor *k* in ANIMO will be 0.1 and 1.0 for slow and fast reactions respectively. No precise timing information was added to the model: e.g., phosphorylation reactions are simply faster than gene expression, but time scales are neither realistic nor precise. This means that the model cannot faithfully predict a particular phosphorylation spike to occur in the first 20 min, nor can it show that a specific gene is expressed within 4 h. What the model does show is that the phosphorylation spike occurs much faster than the gene expression. Because of this absence of timing information, we preferred to avoid time-bound predictions and concentrated only on the steady-state results.
• In the original model, the activity of a node that is not activated is assumed to automatically revert to 0. This assumption is made explicit in ANIMO by adding a self-inhibition loop to each node in the network, with *k* equal to 0.1 or 1.0 depending on the type of reactions influencing the node (i.e., “slow” or “fast”). Due to this self-inhibition, each node will gradually revert to 0 activity in the absence of upstream activations.
• Kinetics that use OR (additive) semantics are translated into independent edges in ANIMO, with interaction strengths balanced to match the original model. For example, node “Ras” is activated from 4 different sources independently (Wnt, BMP, FGFR1, FGFR3), all with strength *k* = 0.444. The self-inhibition of Ras has strength *k* = 1.0, so having any one of those four nodes at activity 100 with all others at 0 will lead to Ras activity 44 out of 100;
• Kinetics that use AND semantics are translated with ANIMO’s AND kinetic scenario, which allows two nodes to influence the activity of a single target. An AND interaction is only active when both upstream nodes are active.
• Reactions involving more complex logic rules were modelled case-by-case, using dummy nodes when necessary to faithfully reproduce the behavior of the original model. As an example, see the dynamics of the Destruction Complex (DC) in Supplementary Figure S2. The nodes “DC dummy,” “DC canonical,” “DC degradation” and their interactions are used to describe the kinetic formula for the node “destruction complex.”
• Some proteins in ECHO need to be both expressed and post-translationally activated to perform their task. In those cases, a three-node pattern is adopted that allows to represent the two processes of protein production and post-translational activation. As an example, consider the dynamics of Sox9 in ECHO: expression and activation of Sox9 are controlled separately through the nodes “Sox9 prot” and “Sox9 PTM” respectively. All influences on Sox9 expression affect “Sox9 prot” with “slow” kinetics, while all post-translational modifications are modelled as influences on “Sox9 PTM” with “fast” kinetics. Finally, Sox9 activity is determined by the “Sox9 prot AND Sox9 PTM activate Sox9” interaction. In this way, Sox9 must be both expressed and post-translationally activated to be activated and exert downstream effects. For several proteins, expression and post-translational activation are regulated separately: AKT, ATF2, CCND1, Dlx5, Ets1, FGFR1, FGFR3, IGF-1R, Lef/Tcf, MEF2C, Msx2, PI3K, PPR, RUNX2, SOX9.

The model originally obtained from the translation contained 120 nodes and 343 interactions (see Supplementary Figure S3), which were simplified by removing the nodes representing expression or post-translational modification processes that are not modelled in ECHO. In particular, the “prom” nodes (which represented promotors on the DNA) have been removed, and the influences on protein production have been redirected to the “prot” nodes instead. The resulting first version of the ECHO model (GP model) contained 92 nodes and 296 edges (See Supplementary Figure S4). For Parameters of Nodes and Edges, see Supplementary Table S3). We took this model as a representation of a growth plate chondrocyte (GP). Through further adaptations (see below) we obtained an articular cartilage model (AC). Initial simulations in which all starting activities of all nodes were randomly initialized revealed three possible stable states: a SOX9-positive (SOX9+) state, a RUNX2-positive (RUNX2+) state, and a NULL state in which neither SOX9^+^ or RUNX2^+^ was reached (see Table 1). Over 90% of all initializations arrive at in a Null state in which all nodes assume the activity value zero (see Table 2). Please note that the SOX9^+^ and RUNX2^+^ states are mutually exclusive.

**TABLE 2:** Distribution of ECHO cell fates from 1,000,000 random initializations for each model. Errors give the boundaries of 99% confidence intervals.

Model	SOX9^+^ (%)	RUNX2^+^ (%)	Null (%)	SOX9^+^/RUNX2^+^
1 (GP)	1.49 ± 0.03	6.95 ± 0.07	91.56 ± 0.07	0.21 ± 0.01
2	0.54 ± 0.02	0.19 ± 0.01	99.27 ± 0.02	2.8 ± 0.3
3	1.78 ± 0.03	0.81 ± 0.02	97.41 ± 0.04	2.2 ± 0.1
4 (AC)	0.61 ± 0.02	0.012 ± 0.003	99.37 ± 0.02	50 ± 13

### Validation of the Model

For validating the predictions, either existing literature or wet lab experiments data can be used. In our case, we wanted to use a system of developing cartilage, hence we selected rat metatarsals from 1-day old rat pups. For interested readers, we would like to refer to (Scholma et al., 2014; Schivo et al., 2016; Khurana et al., 2021) for a systematic method to generate and validate a computational model. We selected combinations of treatments based on the computational model predictions that led to a switch in SOX9 and RUNX2 active state. Previously, it has been shown that a week of treatment is enough for observing changes in longitudinal length and in other parameters of metatarsals, hence we treated the metatarsals with selected molecules for 6 days (Landman et al., 2013). Concentrations of molecules were used as mentioned in (Huang et al., 2001; Zhang et al., 2009; Han et al., 2011; Govindaraj et al., 2019) and described below.

### Metatarsal Culture

Three medial metatarsals per hind leg were carefully dissected out from 1-day old rat pups (Rj Han: WI Wistar rats purchased from Janvier Labs). Animal experiments were approved by Instituut Voor Dierenwelzijn (IVD) at University of Twente. After isolation, metatarsals were individually cultured in 24-well plates in 200 μl/well in Minimal Essential Medium (MEM) α medium supplemented with 10% Fetal bovine serum (FBS), 100 U of penicillin-streptomycin and 1% GlutaMAX supplement for 48 h. After this, 6 metatarsals per treatment were treated with various combinations of the small molecules (H-89 (30 μM), Tanshinone IIA (6 μM), and PD98059 (25 μM), Recombinant human IGF1 (100 ng/ml), Recombinant human BMP7 (100 ng/ml), and Rh PtHrP (100 ng/ml) for 6 days.

### Morphometric and Histological Analysis

Microscopic images were taken at different time points and the longitudinal growth of the bones was measured along the sagittal axis using ImageJ software. For histological examination, metatarsals were fixed in 10% formalin and dehydrated in ethanol series before embedding in paraffin. Five micrometer sections were cut using a rotary microtome (Shandon). The sections were dried for at least an hour at 65°C and stained with Safranin O stain for proteoglycan quantification. The slides were then deparaffinized in 100% xylene twice for 5 min and rinsed in 100% EtOH. A hydration series of 2 × 100, 1 × 96, 1 × 90, 1 × 80 and 1 × 70% EtOH was done, each for 2 min. The sections were then rinsed in demineralized water for 2 min and stained with Gill #3 hematoxylin staining solution for 6 min after which they were washed in running tap water for 15 min. The sections were then stained with Fast Green (0.001 w/v % in dH2O) for 3 min and quickly rinsed with 1% v/v Acetic acid in dH2O for 10 s. After staining with Safranin O (0.1% w/v in dH2O) for 6 min the slides were rinsed 2 times in 70% EtOH for 1 min each and a dehydration series in 1 × 80, 1 × 90, 1 × 96, 2 × 100% EtOH. The slides were then incubated twice for 5 min in 100% xylene after which they were immediately mounted in GLC mounting medium. The slides were kept in xylene while mounting. Slides were counterstained with hematoxylin Gill #3 for 30 s and mounted with GLC™ mounting medium (Sakura). Images were taken using a Nanozoomer (Hamamatsu).

### Quantification of GAG Staining

To quantify the intensity of histological staining ImageJ version 1.51 was used. The images were converted to 8-bit grayscale and the plugin Image Inverter was then applied. The straight-line tool was then used to draw a line the length of the scalebar, and set scale was used to apply a spatial calibration converting units from pixels to mm. In set measurements area, mean grey value and min & max were selected. The polygon selection tool was then used to select the cartilage portion of the metatarsal base, excluding the hypertrophic zone and any tissue stained with fastgreen (such as surrounding fibrotic tissue). Measure was then used to quantify metatarsal base area, and average grey value of the metatarsal base. The rectangle tool was then used to select areas from the background. The average grey value was then measured, and this background value was subtracted from the average grey scale value of the metatarsal base to give an indication of the staining intensity. The straight-line tool was finally used to measure the length of the different zones after setting scale.

### Statistical Analysis

Statistical analysis was performed in R, using the statistics package ggpubr. A Welch Two Sample *t*-test was performed between control and treatment as well as between individual treatments using the code: stat_compare_means (method: “t.test,” comparisons = my_comparisons). Differences were considered significant when *p* < 0.05.

### Reference to Materials

Minimal Essential Medium (MEM) α medium, Gibco/Life Technologies, #22571-020.

Fetal bovine serum, 10%, Gibco/Life Technologies, #10270106.

Penicillin-Streptomycin, Gibco/Life Technologies, #15140-122.

GlutaMAX, Gibco, #35050-061.

H89 (30 μM), Merck, #B1427-5MG.

Tanshinone IIA (6 μM), #T4952-5MG, Merck.

PD98059 (25 μM), #S1177, Selleckchem.com.

Rh IGF1 (100 ng/ml), # 354-BP-010, R and D systems.

Rh BMP7 (100 ng/ml), #100-09, Peprotech.

Rh PtHrP (100 ng/ml), #100-09, Peprotech.

## Results

### Modeling Growth Plate Cartilage Using ANIMO

Chondrocytes, that secretes and shapes the extracellular matrix necessary for the cartilage load-bearing properties, are differentiated from mesenchymal stem cells in a sequence of events following mesenchymal condensation. Chondrogenic differentiation and hypertrophy are directly and tightly regulated by the activity of two main transcription factors. SOX9 is the master transcription factor for chondrogenic development and a key inhibitor of hypertrophic differentiation. RUNX2 is a transcription factor that facilitates hypertrophic differentiation that occurs in the growth plate, and a key factor for osteoblastogenesis during subsequent bone formation (Mackie et al., 2008; Cheng and Genever, 2010). The balance of the activities of these two factors controls the switch between formation of permanent articular cartilage versus transient hypertrophic cartilage in the growth plate (Eames et al., 2004; Zhong et al., 2015). However, the complexity of the signaling network that controls the activities of SOX9 or RUNX2 prevents a thorough understanding of the mechanisms that regulate formation of transient or permanent cartilage.

To investigate the intricate signaling network in cartilage we set out to build a computational model according to logical rules we described previously (Scholma et al., 2014). To start, we used pre-existing Boolean model and additive models of the growth plate (Kerkhofs et al., 2012; Kerkhofs and Geris, 2015; Kerkhofs et al., 2016). Seven signaling pathways known to be important in cartilage development and maintenance: WNT, BMP, TGFβ, IHH, IGF, PTHrP, and the FGF pathways are described. In contrast to Boolean networks, ANIMO is based on activity networks, where activity represents an integrated value that accounts for modulations in gene expression at posttranscriptional and post-translational levels. The rules we used to translate the original model into ANIMO can be found in the Methods. The resulting ANIMO network, which we called ECHO (Executable CHOndrocyte), contains 120 proteins (nodes) and 343 interactions (edges) representing the downstream signaling events that influence SOX9 and RUNX2 (Supplementary Figure S3). For a number of these proteins, expression and post-translational activation are regulated separately (methods: model definitions Supplementary Figure S4). Node activities range with integer values between 0 and 100, while interactions are described as positive or negative influences. Single-parameter simplified kinetics describe the rate at which each interaction influences its target node’s activity.

We defined the network configuration in a stable SOX9-active state as a healthy articular chondrocyte or stable chondrocyte phenotype, whereas a state in which RUNX2 is active is associated to chondrocyte hypertrophy and bone formation. The adaptation of the growth plate gene expression network to a protein activity network in ANIMO is referred to as Model 1. The model enabled us to obtain insight into the activities of the proteins in the network leading to development of stable cartilage (SOX9^+^) or transient hypertrophic cartilage as found in the growth plate (RUNX2^+^).

### A Model of Growth Plate Cartilage Is Adapted Towards Articular Cartilage Based on Global Gene Expression Microarrays of Growth Plate and Articular Cartilage

**FIGURE 1:**
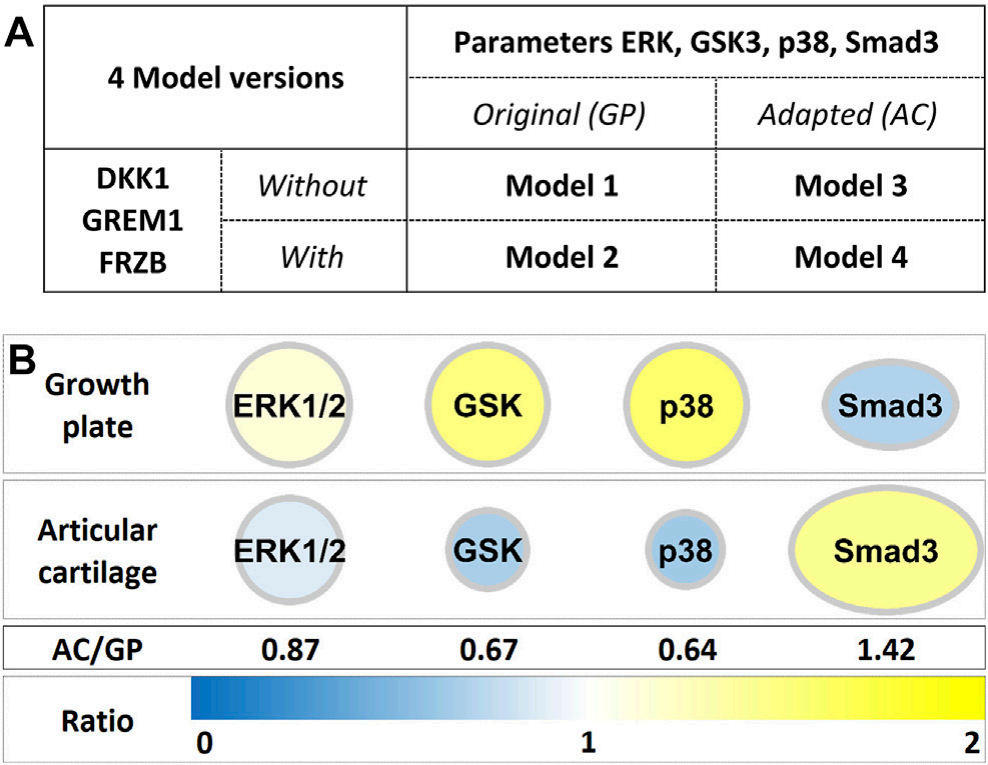
**(A)** Adaptation of ECHO resulted in 4 model versions, depending on the presence or absence of DKK1, FRZB and GREM1 and the changes in parameters of ERK1/2, GSK, p38 and SMAD3. **(B)** Model parameters of ERK1/2, GSK3β, p38 and SMAD3 were adapted to previously found differences in growth plate and articular cartilage mRNA expression (Leijten et al., 2012; Schivo et al., 2019).

Growth plate cartilage and articular cartilage share a common lineage in development (reviewed in Onyekwelu et al., 2009; Goldring, 2012). Many studies have been directed towards identifying specific markers for transient and permanent cartilage (Emons et al., 2011; Gelse et al., 2012; Leijten et al., 2012; van Gool et al., 2012). We identified DKK1, FRZB (WNT antagonists) and GREM1 (BMP antagonist) as the natural brakes on hypertrophic differentiation and regulation of the maintenance of the articular phenotype (Leijten et al., 2012). We therefore incorporated DKK1, FRZB and GREM1 into Model 1 to generate Model 2 (Figure 1A, Supplementary Figure S5) (Schivo et al., 2019).

Differential gene expression analysis between growth plate (GP) and articular cartilage (AC) further indicated subtle but significant differences in the expression of four genes whose corresponding proteins were already represented in our model: ERK2, p38γ, GSK3β and Smad3 ((Leijten et al., 2012), Figure 1). For the other nodes in the network, no changes in gene expression between GP and AC were observed and these are therefore unchanged. To represent the different expression of these four genes in ECHO, we reasoned that the strength of effects directly downstream of these factors is likely to correlate with their expression level and subsequently the protein activity. We therefore multiplied downstream interaction parameters with the relative expression levels to take into account the differences between tissues. For example, the microarray data show that articular chondrocytes express less p38 than cells from growth plate (Figure 1B AC/GP = 0.64). Hence, we adjusted parameters of the interactions downstream of p38 by a factor of 0.64 to yield Model 3 (without DKK1, FRZB and GREM1, but with modification of output parameters for ERK/p38/GSK3/Smad3) and Model 4 (including DKK1, FRZB and GREM1 and modification of output parameters for ERK/p38/GSK3/Smad3) (Figure 1B). To obtain detailed insight into the effects of these adaptations on reaching a state in which either SOX9 or RUNX2 are robustly active (i.e., SOX9^+^ or RUNX2^+^ states), all *in silico* experiments were carried out for all four models (Figure 1A).

As a first assessment of the properties of the four models, we performed Monte Carlo simulations in which all nodes are initially assigned a random, uniformly distributed activity level over the entire range of theoretical values (i.e., the interval [0, 100]). Each initialized model is then simulated until a stable state is reached. Analysis of the results of 1,000,000 simulations for each model shows that three distinct stable states are possible for ECHO Models 1–4 (Table 1). Over 90% of all initializations arrive at a Null state in which all nodes assume the activity value zero. This result is attributable to the fact that in ECHO protein activities are programmed to taper off and reach baseline in the absence of upstream activating factors. Only initializations of essential network components that have activity patterns above certain threshold levels will escape from returning to the Null state.

**FIGURE 2:**
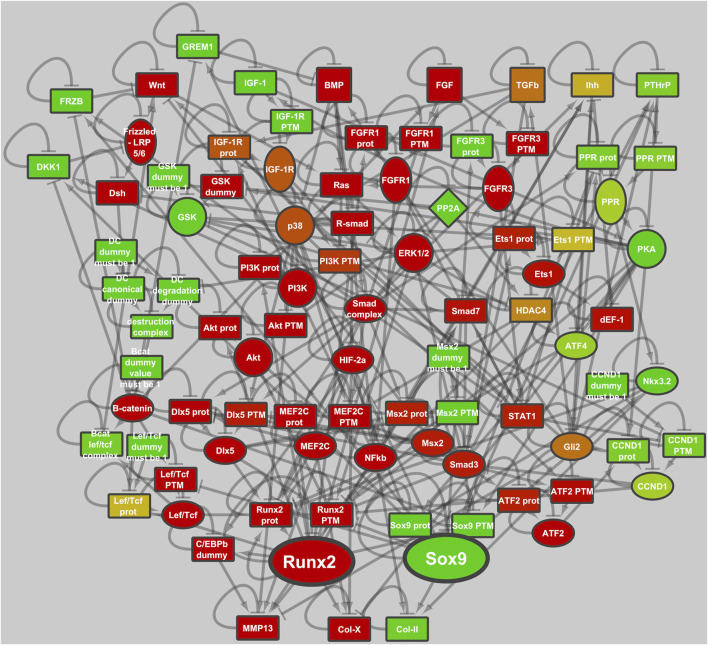
ECHO, executable chondrocyte, describes the development and maintenance of articular chondrocytes. The activities of the transcription factors SOX9 and RUNX2 are regulated by an intricate network of signal transduction pathways, including IHH, PTHrP, FGF, WNT, BMP, TGFβ, HIF and IGF. The model is depicted in the SOX9+ state and node. Activity is represented on a scale from red (inactive) via yellow to green (active).

More interesting from a biological perspective are the other two stable states that are either SOX9^+^ and RUNX2^+^. In Model 1 (the original GP model), the RUNX2^+^ cell fate is about 5 times more likely to occur than the SOX9^+^ cell fate, Table 2. Addition of the genes that were highly expressed in articular, but not in growth plate cartilage, DKK1/GREM1/FRZB (Model 2, Supplementary Figure S5), causes an increase in the Null state as expected, because those factors repress WNT and BMP signaling, thus decreasing the fraction of initializations capable of escaping the Null state. The RUNX2^+^ state is much more affected by DKK1/GREM1/FRZB than the SOX9^+^ state, and the latter becomes dominant. Adaptation of parameter settings for ERK/p38/GSK3 (Model 3), to better represent protein concentrations in articular chondrocytes, causes a decrease in the RUNX2^+^ fate, while the adaptation of SMAD3 further increases the fraction of SOX9^+^ fate. The two adaptations together (addition of DKK1/GREM1/FRZB, and adaptation of the parameters downstream of ERK/p38/GSK3/SMAD3, Model 4, Figure 1) virtually eliminate the RUNX2+ fate. In this respect, its behavior resembles that of articular cartilage, which is under stable control of SOX9. In the remainder of the paper, we will consider Model 1 (Supplementary Figure S4) to be a growth plate cartilage model (ECHO GP), while Model 4 (Figure 2) represents an articular cartilage model (ECHO AC).

### Constitutive Activation and Knock-Out of Individual Nodes in the Network Provides Information on the Role of Proteins in Determining Cell Fate

**FIGURE 3:**
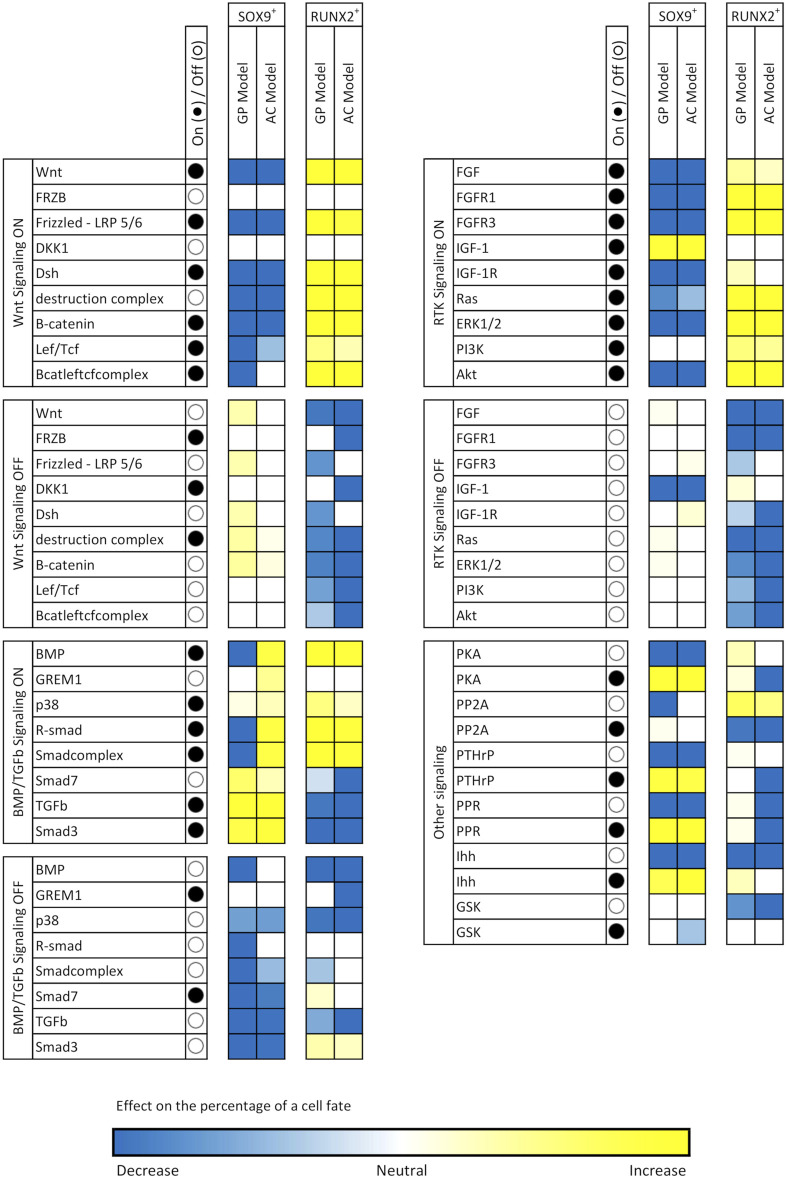
Effects of node perturbations on cell fate. Each node in ECHO was set as either constitutively active (activity fixed at 100, ●) or knocked-out (activity fixed at 0, ○), while all other nodes were randomly initialized over the course of 10,000 simulations. The resulting percentages of cell fates were computed and compared with the percentages in Table X1. The colors the cells in this table show the magnitude of deviation from the non-perturbed values and give an indication of the importance of a node for a cell fate.

To understand the role of each node in determining cell fate, we individually perturbed the activity of the nodes by fixing their activity to either 0 (*in silico* knock-out, K.O.) or at 100 (constitutive activation). The other nodes were randomly initialized over the course of 10,000 simulations and cell fate distributions were compared with the unperturbed situation (Table 2) to assess the influence of each perturbation (Figure 3). Given the bi-stable behavior of ECHO, one can expect that the effects of perturbations follow three intuitive “rules”: 1) If a node is more active in the SOX9+ fate than in the RUNX2+ fate, then activation of this node will favor SOX9+ cell fate and knockout will favor RUNX2+ cell fate, and vice versa. 2) If a knockout or activation favors a specific cell fate, it is detrimental to the other fate. 3) If the knockout of a node favors a specific cell fate, activation of the same node is detrimental to this fate.

WNT is more active in the RUNX2^+^ fate (activity 100) than in the SOX9^+^ fate (activity 29 in the GP model, and 0 in the AC model) and is an example that follows all the rules above. Interestingly, a node that has activity 0 in a cell fate can still affect the probability of reaching that fate when it is knocked out. An example is PP2A, which has activity 0 in RUNX2^+^. Knockout of PP2A increases the RUNX2^+^ state from 7 to 44%. This happens because PP2A inhibits ERK, which in turn activates RUNX2; thus: knocking out PP2A (indirectly) activates RUNX2.

R-SMAD is an exception to rule 2, as a constitutively active R-SMAD in the AC model makes both SOX9^+^ and RUNX2^+^ percentages rise significantly. In other cases, both activation and K.O. of the same node have similar effect on a cell fate, contravening rule 3. For example, keeping BMP inactive in the GP model annuls the chance to reach a SOX9^+^ state, and the same effect is observed if BMP is kept at 100% activity. Interestingly, this does not occur in the AC model. These complex effects are also known in the wet-lab, where BMP2 has a dose-dependent effect on stem cell differentiation, and can stimulate both cartilage and bone formation (Wang et al., 1993). Another interesting exception to rule 3 is TGFβ: although it is more active in a RUNX2^+^ state, its constitutive activation significantly increases the chance of reaching a SOX9^+^ fate and prevents reaching RUNX2^+^.

These complex effects recapitulate experimental findings, where BMP2 has both transient and permanent dose-dependent effects on stem cell differentiation, and can stimulate both cartilage and bone formation (Wang et al., 1993). TGFβ is also interesting because this signaling ligand is more active in a RUNX2^+^ state, but its constitutive activation significantly increases the chance of reaching a SOX9^+^ fate, while preventing a RUNX2^+^ state.

Although the role of these proteins in relaying information to the nucleus is known, knockout or constitutive activity for most of these proteins in determining cell fate has not yet been described. Published KO animal experiments (Yoon et al., 2005; Jing et al., 2013; Jing et al., 2014; Chen et al., 2015) validate the network topology and parameter settings for ECHO. Our findings indicate that computational models offer rationale outputs that can inform new *in vivo* and *in vitro* experimentation to elucidate the molecular mechanisms governing cartilage and bone development.

### Using Literature to Validate Topology and Dynamics of ECHO

In any computational model one wonders how much the model represents biological situations with respect to the time dependency of reactions and the topology of the model. Of course, the Chinese proverb “Life is like an echo: What you send out usually comes back to you.” could be quoted here, since the model predictions should reflect the data that were put in. For building ECHO, we did not use any model training and based the topology of the cross-talk interactions of the various pathways on different studies. It is therefore still useful and necessary to validate whether the results of the *in silico* experiments in ECHO reflect the literature. We therefore investigated whether K.O. animal experiments that were not used for the model building, validate the network topology and parameter settings for ECHO. We aimed to validate some of the data of our KO and overexpression experiments of Figure 3. Quite a few papers discuss the double role of BMP in articular cartilage as well as in chondrocyte hypertrophy and OA. We also observe this in the model, where we find that the role of BMP is concentration dependent. In conditional BMPR1a KO mice, the lack of Bmpr1a leads to significant chondrodysplasia and almost eliminated the chondrocyte phenotype with decreased SOX9, collagen II and proteoglycan (Yoon et al., 2005; Jing et al., 2013; Jing et al., 2014). In ECHO, BMP2 affects both RUNX2 and SOX9, which is also seen in BMP2/4 double KO embryos where SOX9, ACAN and collagen type II (COL2A1) mRNA levels were reduced and RUNX2 protein expression was reduced in the proliferating and pre-hypertrophic areas (Shu et al., 2011; Liao et al., 2014). In addition, Shu et al. found that BMP2 induces RUNX2 expression at both transcriptional and post-transcriptional levels (Shu et al., 2011). We showed in ECHO that PTHrP and IHH overexpression increased the occurrence of stable SOX9 states, while KO reduced the SOX9 phenotype. Indeed, in IHH KO mice it was shown that expression of SOX9 and RUNX2 as well as PTHrP was low and growth was inhibited in the temporomandibular joint, indicating that IHH is indispensable for proliferation and expression of transcriptional regulators such as RUNX2 and SOX9 (Shibukawa et al., 2007; Ochiai et al., 2010).

This indicates that using a computational model offers advantages to biologists that now depend on many mouse models to elucidate the molecular mechanisms governing cartilage and bone development.

### Perturbation of Pairs of Nodes in the Network Reveals New Pathways That can Be Manipulated for Future Cartilage Disease Therapy

**FIGURE 4:**
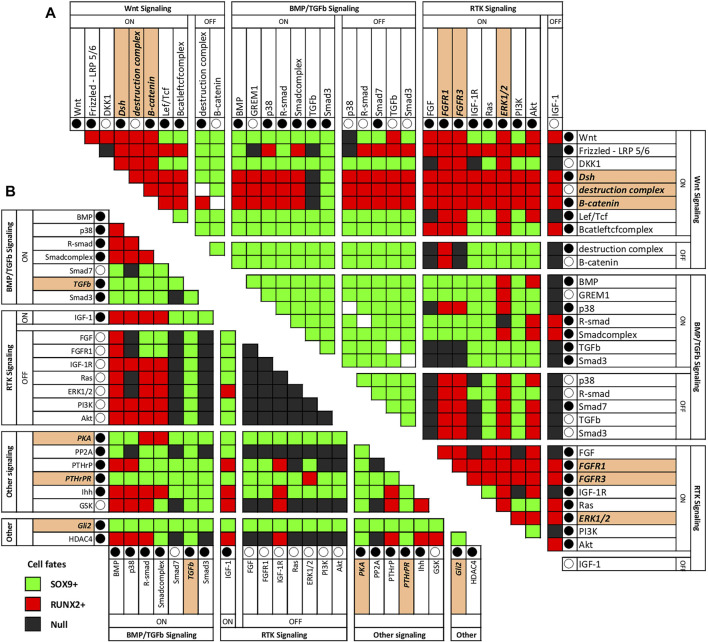
Combination of perturbations that cause a SOX9+ to switch to a RUNX2+ state **(A)** or RUNX2^+^ state to switch to a SOX9^+^ state **(B)** in the AC model. This figure shows combinations of knockout and overexpression that induce switches between the RUNX2 and SOX9 positive states. An overview of all combinations of knockouts and overexpression can be found in the supplemental material. Each pair of nodes in the network was perturbed in all combinations of knock-out (○) and constitutive activation (●), while all other nodes were initialized as in the RUNX2^+^ state. After one simulation, the resulting stable state was recorded. Single node modifications are highlighted if they can be used alone to obtain the switch. **(A)** Switch from SOX9^+^ to RUNX2^+^, **(B)** Switch from RUNX2^+^ to SOX9^+^. The extended figure can be found in the Supplementary Figure S6.

Corresponding to the biological reality of cartilage diseases, such as osteoarthritis, OA, in the AC model a switch from the SOX9^+^ to the RUNX2^+^ state is possible (Zhong et al., 2016a). This allows interrogation of the model for conditions that cause a switch to RUNX2^+^. Such conditions in the model could recapitulate changes taking place in OA patients. Even more interesting from a therapeutic perspective are interventions that could reverse such a switch. We performed all-or-nothing perturbations of all combinations of two nodes in the network to find such conditions. A summary of the results showing combinations of knockouts and/or overexpression that induce switches between the RUNX2 and SOX9 positive states is given in Figure 4. The complete analysis, also including nodes for which knockout or overexpression had little to no effect on the state is shown in Supplementary Figure S6.

There are nodes whose activities are linked with a switch from a SOX9^+^ stable state to a RUNX2^+^ state. These nodes could indicate mechanisms by which healthy articular cartilage undergoes hypertrophy to become transient cartilage as occurs in a subset of OA patients (Gelse et al., 2012; van der Kraan and van den Berg, 2012; Zhong et al., 2015). Activation of the WNT or FGF signaling pathways results in a switch from SOX9^+^ to RUNX2^+^ (Figure 4A). This is not unexpected, as both WNT and FGF signaling have been related to induction of hypertrophy in cartilage (reviewed in (Zhong et al., 2015)). Combinations of factors that induce a switch from SOX9^+^ to RUNX2^+^ are simultaneous activation of WNT3a and PKA (but addition of only WNT3a already induces a RUNX2+ state), DLX5 and inhibition of PKA, combination of anti-DKK1 and anti-FRZB (already described in (Zhong et al., 2016b)), and inhibition of IGF via GLI2 and ERK. Since we could not find literature on the combination of DLX5 and inhibition of PKA, we decided to further investigate this.

Inversely, in addition to SOX9 activation, there are combinations of factors that are sufficient to cause a transition of the RUNX2^+^ state to the SOX9^+^ state in the AC model: simultaneous addition of BMP7 and PTHrP, addition of TGFβ (alone), simultaneous addition of IGF1 and PTHrP, and simultaneous inhibition of ERK in combination with addition of IGF. Using model-checking we further investigated some of these combinations.

### Using Model-Checking to Test and Refine Candidate Treatment Conditions

Among the combinations shown in Figure 4, we selected a series of interesting treatment conditions that could be tested in laboratory. Treatment conditions for which sufficient literature was available, were omitted. Table 3 shows the treatment conditions that were selected as most promising candidates.

**TABLE 3:** Model checking was performed on a selection of combinations of nodes that were predicted to switch cell fate to a preferred SOX9+ fate or a RUNX2+ state. All treatments were tested using both the SOX9+ and RUNX2+ initial states. “No switch” means that the initial state is constantly preserved, i.e., that the property “[initial state] must persist indefinitely” is true. “Always possible” means that the model is guaranteed to switch state, i.e., the property “[opposite state] can always be reached” is true.

Treatment #	Target 1	Target 2	Preferred state	Model checking
1	No treatment	No treatment	N/A	SOX9+: No switch
				RUNX2+: No switch
2	ERK OFF	IGF1 (later) ON	SOX9+	SOX9+: No switch
				RUNX2+ → SOX9+: Only with later addition of IGF1
3	PTHrPR ON	BMP7 ON	SOX9+	SOX9+: No switch
				RUNX2+ → SOX9+: Always possible. Note: We are adding PTHrPR (PPR). PTHrP addition is not strong enough.
4	DLX5 ON	PKA OFF	RUNX2+	SOX9+ → RUNX2+: Always possible
				RUNX2+: No switch

**FIGURE 5:**
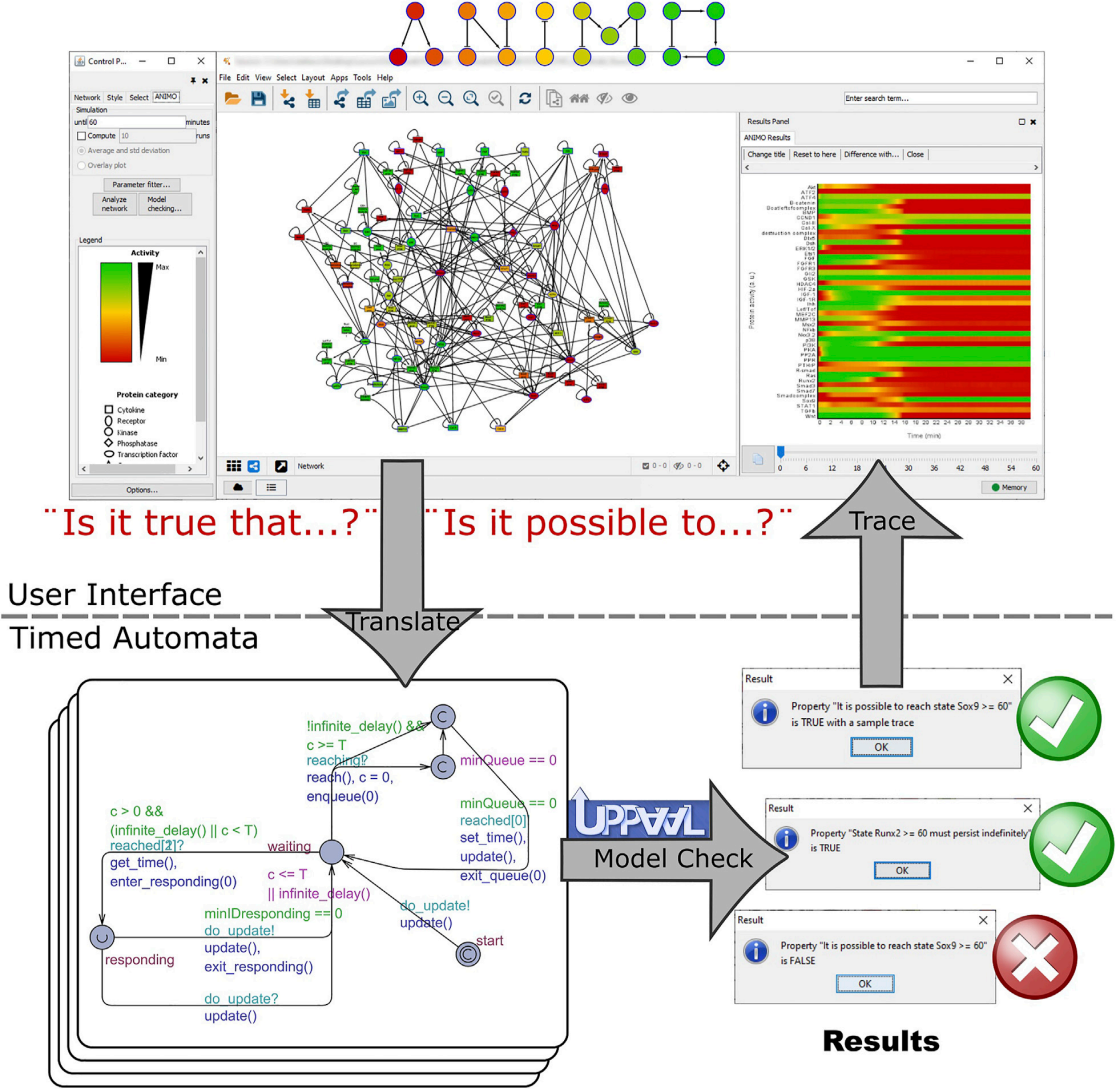
Workflow of the model checking experiments. Model checking is used to ensure that the predicted behavior based on one simulation run is not due to errors or artifacts of the model. The tested conditions were: 1. BMP7+PTHRP, 2. ERK inhibition and addition IGF1, and 3. DLX5 activation with inhibition of PKA off. We used model checking to ensure that switches occur in the model as expected. If a formula of the type “[initial state] must persist indefinitely” is found to be true, no further action is taken: we have the guarantee that the property is true in all possible future evolutions of the current configuration of the model. However, if the formula is found to be false (or equivalently, the opposite formula “[opposite state] can be reached at least once” is found to be true), an example trace is automatically produced by the UPPAAL tool, analyzed by ANIMO and represented as an activity graph in Cytoscape. This trace runs through one possible execution run of the model and illustrates how it is possible that the tested property becomes false (resp. true).

For each of the selected treatment conditions, we used model checking to ensure that the predicted behavior based on one simulation run was not due to errors or artefacts of the model. The formal technique of model checking allows to automatically test all possible behaviors of a model against a given property. At the end of the analysis, the property is found to be either true or false for the given model (see Figure 5), providing a guarantee that cannot be obtained by just observing one single simulation run. Because model checking is computationally intensive, we could apply it only to a restricted set of conditions.

Each of the selected treatments was tested in ANIMO using the model checking feature, starting from both a SOX9+ and RUNX2+ initial state. In case no change was expected (for example, if we start from a RUNX2+ state and we expect that no switch occurs), we tested a query such as “The state RUNX2+ must persist indefinitely,” which is automatically translated by ANIMO into the formal language CTL as “A [] RUNX2 >= 60 && SOX9 < 20” (= “node RUNX2 has always an activity of at least 60% while node SOX9 never reaches 20% activity, no matter what happens in the model”). This is automatically tested with the model checker UPPAAL, and the result (True/False) is shown to the user. In case a change was expected, we tested a property such as “It is always possible to reach the state SOX9+,” which was translated into “A<> RUNX2 < 20 && SOX9 >= 60” (= “it always guaranteed that we reach a configuration in which node RUNX2 has less than 20% activity while node SOX9 has at least 60% activity”). If a change can occur only in some cases, while in others no change is obtained, both the previous properties are found to be false. In this situation, a property such as “It is possible to reach state SOX9+” (translated into “E<> RUNX2 < 20 && SOX9 >= 60,” i.e., “it is possible, but not guaranteed, to reach a configuration in which node RUNX2 has less than 20% activity while node SOX9 has at least 60% activity”) is True, and ANIMO additionally shows a trace (plot of the node activities) as a proof that the requested state “SOX9+” can indeed be reached.

It is interesting to note that in one case the expected a result shown in Table 3 is not coherent with what the model results show in Figure 4: for treatment number 2 (ERK OFF, IGF1 ON) the model shows that a RUNX2+ configuration does not switch to SOX9+ (Figure 4), and this was confirmed with model checking. Indeed, the query “It is possible to reach the state SOX9+” (in CTL: “E<> SOX9 >= 60 && RUNX2 < 20”) evaluates to false, which means that it is never possible to obtain a SOX9-positive activity state starting with the given configuration. We note that the only reachable configuration is Null, where both SOX9 and RUNX2 (as well as most nodes in the network) are at 0 activity. However, with some further investigation, we were able to observe that a different timing in the treatment actually can lead to a switch to SOX9+. We modified the model such that the addition of IGF1 does not occur immediately: instead of being completely active from the beginning, IGF1 can be added at a later, purposefully left unspecified, point during the evolution of the model. We then performed a model checking query to see whether there is at least one way to reach a SOX9+ state (“It is possible to reach the state SOX9+,” “E<> RUNX2 < 20 && SOX9 >= 60”): indeed, there is. However, the query “It is guaranteed that a SOX9+ state occurs” (“A<> RUNX2 < 20 && SOX9 >= 60”) evaluates to false, because not all timing choices for the addition of IGF1 can lead the model into a SOX9+ state. From the first query, we gather that the addition of IGF1 needs to occur later during the evolution of the model. From the second we obtain that the addition should not come too late, otherwise the model ends in a Null state. As the concept of time is present in ECHO only in a very abstract way (“fast” vs. “slow” reactions), we concluded that the knock-out of ERK causes some adjustment on the signaling (“fast”) parts of the model, which need to be completed before the addition of IGF1 can have the wanted effect and activate the transition to SOX9+. Waiting too much before adding IGF1 can lead to an unrecoverable situation, with the model switching to the Null state instead.

It is also worth mentioning that Treatment number 3 (addition of PTHrP and BMP7) has a different effect in the model than what is intuitively expected. In particular, if ECHO starts in the RUNX2-positive state, the addition (overactivation) of PTHrP and BMP does not directly cause a switch to the SOX9-positive state. This happens because in the RUX2+ state the “PPR Prot” node (which represents the presence of PTHrP receptors) is not sufficiently active: this basically means that the PTHrP pathway cannot be activated unless enough receptors are produced first. Indeed, in Figure 4 the node that by itself can cause a switch from RUNX2+ to SOX9+ is PTHrPR (written as PPR in Figure 2), which is the node representing both the presence and activation of the PTHrP receptor. In reality, we expect that treatment of metatarsals with BMP7 will show enhanced cartilage formation, independent of the PTHrP stimulation (Haaijman et al., 1999).

Model checking of the condition where DLX5 is active and PKA is inactive indeed confirmed the previous finding that AC-model in SOX9 state would switch to a RUNX2+ state.

### ERK Inhibition and IGF1 Overactivation Leads to Increased Bone Growth and Good Cartilage Formation

**FIGURE 6:**
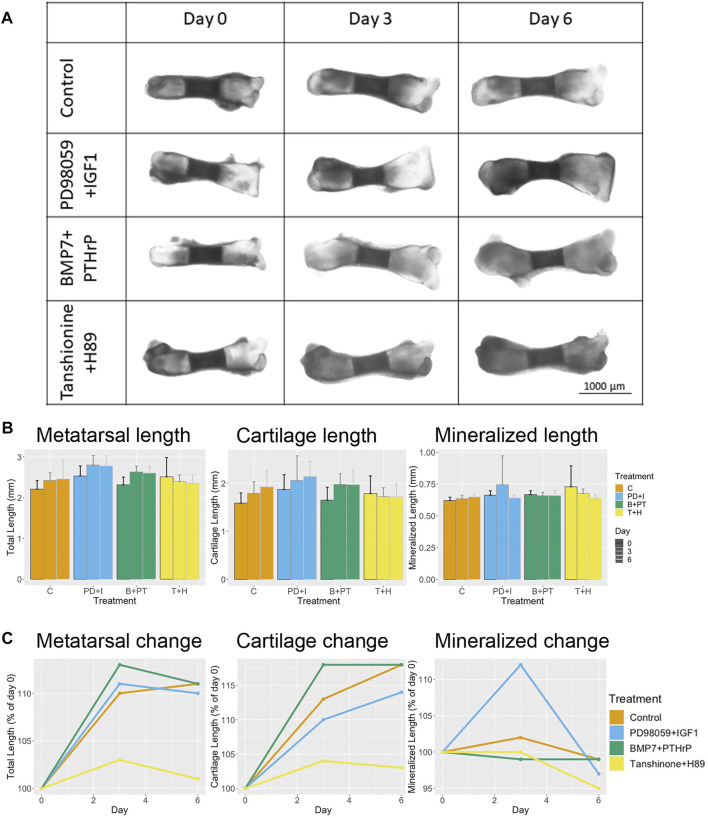
Treatment with a combination of PD98059 (ERK1/2 inhibitor) +IGF1 or a combination of BMP7+PTHrP slightly increases longitudinal length with time, whereas the combination of Tanshinone (DLX5 activator) and H89 (PKA inhibition) decreases longitudinal length as compared to control. **(A)** Morphological changes of representative rat pup metatarsals caused by PD98059 (ERK inhibitor) +IGF1, BMP7+PTHrP and Tanshinone IIA (DLX5 activator) + H89 (PKA inhibitor) at day 0, 3 and 6. **(B)** Change in metatarsal longitudinal length, cartilage length and mineralized bone area length at day 0, 3 and 6 of control and treated samples **(C)**. Percentage change in metatarsal length, cartilage length and mineralized bone length as compared to control. Data represent the mean of at least 6 metatarsals for each condition.

For testing the predicted switch from RUNX2 to SOX9 active state with a combination of ERK inhibition and IGF addition, metatarsals were treated with combination of PD98059 (ERK inhibitor) and recombinant IGF1 for 6 days. For macroscopic validation, longitudinal bone length, total length of both cartilage area and length of mineralized bone area were measured. As per the prediction by the model, it was expected that there would be an increase in the cartilage area growth and inhibition of the mineralized bone area growth over time. Indeed an increase in longitudinal metatarsal length was already observed by day 3 and slight length decrease was observed by day 6 (Figures 6A,B). However, as compared to control the fold change in longitudinal length was lower than that of the control samples at day 3 (not significant, Figure 6C). Increase in cartilage length was observed with time, however, the change was always lower than that in the untreated control. Furthermore, an increase in mineralized bone area was observed at day 3 which was decreased at day 6 even lower than control levels (Figures 6B,C).

**FIGURE 7:**
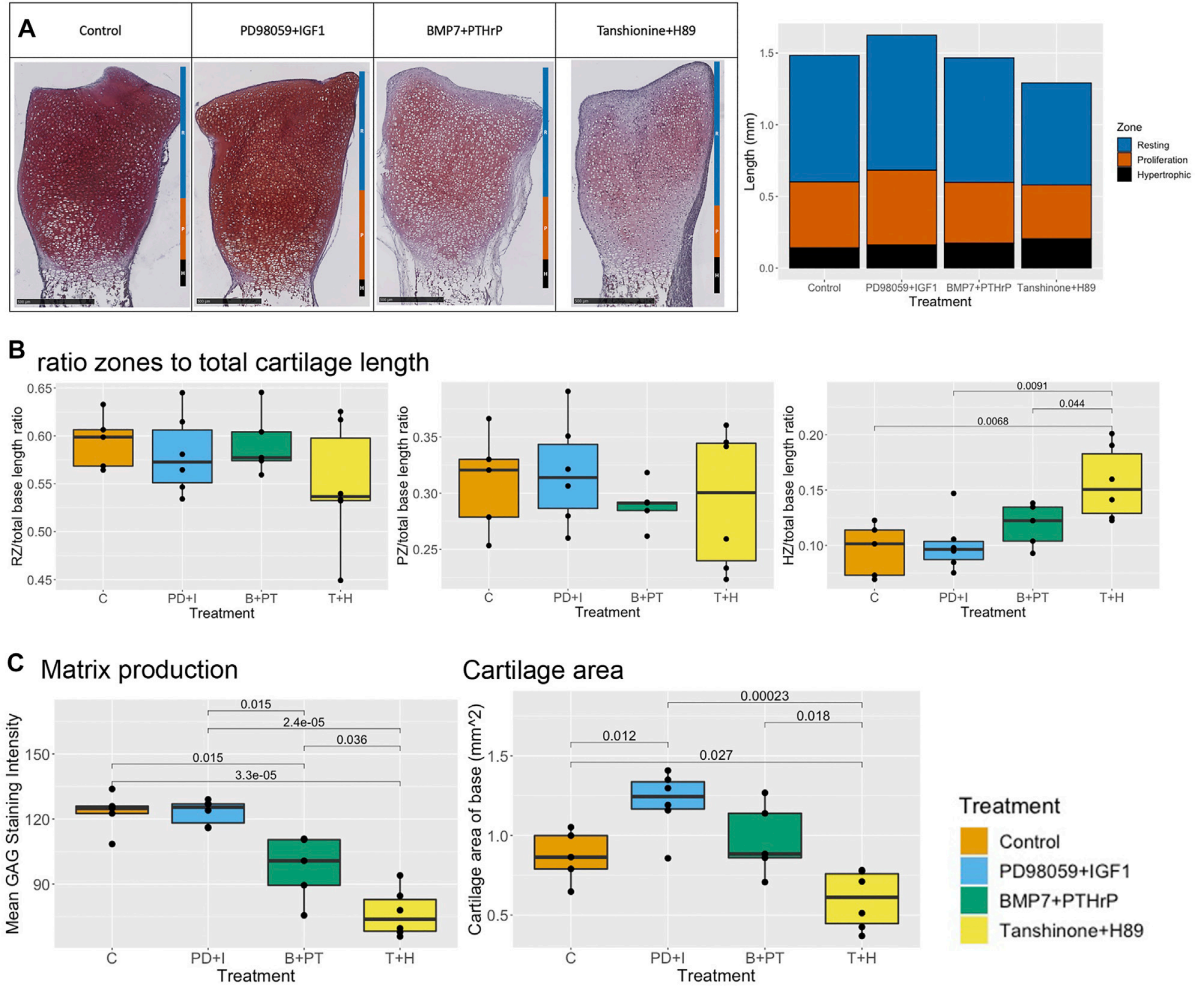
Histological analysis of zonal length, ratio of zones to total cartilage length, proteoglycan production and cartilage surface area in base of rat pups metatarsals **(A)**. Representative Safranin O stained sections of control, PD98059 (ERK inhibitor) +IGF1, BMP7+PTHrP and Tanshinone IIA (DLX5 activator)+H89 (PKA inhibitor) treated rat pups metatarsals at day 6 (left) and comparison of size of resting zone, proliferative zone and hypertrophic zone of stained samples (right) **(B)**. Comparison of ratio of size of resting zone (left), proliferative zone (middle) and hypertrophic zone (right) to total cartilage length of base of metatarsals **(C)**. Comparison of matrix production and cartilage surface area, Welch Two Sample *t*-tests were performed and differences were considered significant when *p* < 0.05.

Microscopically, the validation of the effect of PD98059 and IGF1 on the SOX9 active state was tested by determining the length of the resting, proliferative and hypertrophic zones of Safranin O-stained mid-sagittal sections of metatarsals cultured up to 6 days (Figure 7). It was expected that there would be better cartilage matrix formation, with increased proteoglycan production and a decrease in the size of the hypertrophic zone. Even though the staining intensity was strong, there was no significant difference in the staining intensity as compared to untreated control (Figure 7A). This indicates that overall proteoglycan production was not further enhanced in the presence of these molecules (Figure 7C). A significant increase in cartilage surface area was observed at day 6 as compared to the control (Figure 7C). Interestingly, an increase in the length of the proliferation and resting zones was observed as compared to control (not significant) even though there was a decrease in the cartilage area length as compared to control (Figure 7A). In addition, the length of the hypertrophic zone remained unchanged as compared to the control. Overall, the base of PD98059+IGF1 treated metatarsals was composed of large resting zones, a relatively small proliferation zone and even smaller hypertrophic zone, indicating differentiation towards a SOX9 active state (Figure 7B).

### BMP7 and PTHrP Overexpression Leads to Increased Bone Growth but Poor Cartilage Formation

Another combination of molecules was tested: overexpression of BMP7 and PTHrP. For this purpose, metatarsals were treated with a combination of BMP7 and PTHrP. For this combination, we observed an increase in longitudinal metatarsal length and total length of cartilage at all time points (Figures 6A,B). The fold-change for both parameters was higher than that of the control at day 3, but similar to control samples at day 6 (Figure 6C). In addition, a slight increase in cartilage surface area was observed as compared to control at day 6 (Figure 7C). A decrease in mineralized bone length as compared to the control was observed at day 3, but it was restored to initial levels at day 6 (Figure 6C).

In contrast to the macroscopic parameters as well as ECHO predictions, a decrease in staining intensity was observed as compared to control. Despite the decreased staining intensity, no changes were observed in length of the hypertrophic, proliferative and resting zones as compared to the control (Figure 7A). Overall, the base of BMP7+PTHrP bones was composed of a large resting zone, small hypertrophic zone, and even smaller proliferative zones (Figure 7B). Additional simulations in ECHO verified that addition of PTHrP in the AC-model when in the RUNX2 state, did only activate PKA to about 20%, which is not enough to make a switch to SOX9+. However, ECHO predicts partial node activity for Collagen 2, which may explain the low matrix production observed in these samples. Manual manipulation of the PKA activity to 100% immediately switched the cell fate to SOX9+ (data not shown). This corresponds to what was shown before in Figure 4B, where both PPR and PKA have the power to switch cell fate if fully active.

### Combination of DLX5 Activation and PKA Inhibition Indicate Poor Cartilage Formation

For testing the switch from a SOX9 active state to RUNX2 active state, a combination of DLX activation and PKA inhibition was used. Metatarsals were treated with a combination of Tanshinone IIA (DLX activator) and H89 (PKA inhibitor). An increase in mineralized bone growth was expected. Interestingly not much change in longitudinal bone growth and cartilage length was observed over time (Figures 6A,B). However, a decrease in fold-change of longitudinal bone growth and total cartilage length were observed as compared to control (Figure 6C). In contrast to the prediction, no increase in mineralized zone was observed at these time-points. Surprisingly, the fold-change in mineralized zone was lower than that in the control (Figures 6B,C). Even though the total cartilage length was not significantly changed, a significant decrease in cartilage surface area was observed with time (Figure 7C).

A significant decrease in staining intensity was observed as compared to the control as well as to the other treatments, indicating reduced matrix production. In addition to the decreased staining intensity, the relative length of the proliferative and resting zones was decreased, while the relative length of the proliferative zone was significantly increased as compared to control (Figure 7A). Overall, metatarsals treated with these molecules had the largest hypertrophic zone as compared to control as well as other treatments (Figure 7B), indicating a switch to a RUNX2 positive state as predicted by ECHO.

## Discussion

### Modeling in Biology

Signaling networks are traditionally represented as static graphs. However, in the past years it has become obvious that the temporal and spatial information in these networks confers important dynamic behavior. As static networks do not allow quick modifications to test hypotheses or to include novel findings, a more widespread use of interactive exploration of biological networks and their dynamics could cause a paradigm shift in our understanding of biological networks. To support this shift, we developed ANIMO (Analysis of Networks through Interactive Modeling (Schivo et al., 2012, 2014b; Schivo et al., 2016). ANIMO is a computational modeling tool that enables executable modeling of network dynamics in order to mimic biological phenomena *in silico*. We present here a versatile modeling tool with a low experience threshold that can be implemented used by investigators without formal mathematical training in systems biology and that is based on the intuitive graphic interface offered by Cytoscape. In addition, ANIMO has the ability to predict biological responses, both by manually testing hypotheses, as well as by using the model-checking capabilities offered by the underlying mathematical language UPPAAL (Bartocci et al., 2009; David et al., 2011).

In ANIMO we generated ECHO, Executable CHOndrocyte based on previous models (Kerkhofs et al., 2012; Kerkhofs and Geris, 2015; Kerkhofs et al., 2016). Kerkhofs has shown that these large-scale models can be used to correctly capture the gene expression network dictating chondrocyte hypertrophy in the growth plate (Kerkhofs et al., 2016). Using ECHO, we were able to simulate knock-out and overexpression of all individual nodes in the network. This is something that can only be achieved using computational models, as performing these experiments in the wet-lab is both time consuming and very costly. Moreover, *in silico* experiments provide information on the changes in activity of all nodes in the network upon virtual KO mutations. These very important experiments provide information about the potential roles of, for example, miRNAs for targeting specific factors in the network.

The interesting aspect of these types of experiments is that *in silico* experimentation provides information on all possible combinations and concentrations of the growth factors and cytokines represented in our model. These experiments will provide information on the activities of all biological entities in our model at any time-point after stimulation. This is impossible to achieve in wet-lab experiments and provides detailed description of the biological system at hand.

Even though our computational model is a simple and reusable tool to understand the complex mechanisms behind the switch between SOX9 and RUNX2 activities, certain boundaries are used. Firstly, the model is semi-quantitative, i.e., its numbers do not necessarily reflect reality (in a linear scale), and the concept of “time” in the model can only be seen as a generic sequence of events (minutes/hours in this model have little to no meaning). This is also due to our choice of simplifying the k-parameters to the two main categories of “slow” (transcription+translation) and “fast” (post-translational modifications). Node activities themselves are thus “just numbers,” so while we can see that an activity level of 100 is higher than 10 and interpret this as “high (er) activity” for that node, we cannot define a correspondence between activity levels in the model and protein concentrations in the lab. Another thing to keep in mind is that the *a priori* network topology and choice of nodes in the network is based on existing literature and that means that there is an over-representation of nodes/pathways that are well described in literature. However, the *in silico* experiments are in line with the findings in literature and we therefore feel that the models describe the system well. Also due to the computation restraints, it is not efficient to make large models, so we choose to generate models that are simplified versions of complex networks. Hence, there is an intricate balance between a computational model describing processes in large detail and its prediction ability. Finally, when building a computational model, it is always necessary to find the proper balance between truthfulness (i.e., precision, closeness to reality) and simplicity (abstraction from reality). We refer the interested reader to (Mader et al., 2007; Schivo et al., 2016; Waters et al., 2021). Overall, the precision of a model depends on availability of data and computational tools and the need for detailed information and one should be aware that the final outcome of the computational model depends on these factors.

While computationally efficient models are usually less detailed, they are, still, a great tool to understand network behavior by just using the most important signaling molecules. Computational models can be utilized as a tool to understand the behavior of network especially, in this age, where we have high amounts of proteomic and genomic data available, which will take years to be utilized and validated by wet-lab experiments, especially with regard to cell signaling pathways. Previously, we found that model built with simplified network are sufficient to predict the dynamics and cell fate and thus, can help in prioritizing the wet lab experiments (Schivo et al., 2016; Schivo et al., 2019). This prioritization is what we show in our current publication.

### ECHO as a Predictive Model for Therapeutics and Tissue Remodeling

From a tissue engineering and/or therapeutic standpoint, we wanted to know if it is possible to switch cell fate through perturbation of any combination of 2 nodes in the network. It is experimentally challenging to test these predictions in human primary cells or tissue. This is especially the case for the switch of a RUNX2+ to SOX9+ cell fate, since epigenetic regulation has likely occurred in the process of osteoarthritis (reviewed in Ramos and Meulenbelt, 2017). The resulting methylation of cartilage specific genes, such as SOX9, will therefore prevent the actual switch from a RUNX2+ to a SOX9+ phenotype in OA cells. However, this strengthens the argument of using computational modeling, since it allows us to simulate osteoarthritis development, and as such provides insight into the molecular boundaries that define therapeutic efficacy.

In addition, the combination of factors tested in our *in silico* experiments have individually been described to have a role in cartilage development and in OA, both by cell and in knock-out animal studies (There are many excellent papers, but for more information we refer to these excellent reviews (Kronenberg, 2003; Mackie et al., 2008; Kozhemyakina et al., 2015; Welting et al., 2018 and references herein). However, the combined effects of these factors have not yet been conclusively shown in tissue engineering strategies or therapies. It is therefore likely that when designing therapies for treatment of cartilage defects multiple factors will have to be targeted to get the desired response.

In this study, we make use of metatarsals that are cultured *ex vivo* for validating our model predictions in the wet-lab. We prioritized the model predictions and limited the wet-lab validation to 3 conditions that we compared to an untreated control.

For the treatment consisting of inhibition of ERK with addition of IGF (treatment 2) the model predicts a SOX9+ state. In contrast, ECHO predicts that activation of DLX5 + inhibition of PKA (treatment 3) will switch the cell-fate towards a RUNX2 state.

We assessed the effect of the treatments using various output measures, including size of the metatarsal, as well as the cartilage and mineralized areas. In addition, we assessed extracellular matrix formation by staining glycosaminoglycans in histological sections and measuring the distribution of the different cartilage zones, the hypertrophic, the proliferative and the resting zones. Overall, we see that activation of DLX5 with simultaneous inhibition of PKA leads to inhibition of growth of the metatarsal, a smaller cartilage area, a decrease in matrix production, and a significant increase in cartilage hypertrophy. This may have been partially expected, since DLX5 was shown to regulate osteogenesis in differentiating MSCs (Heo et al., 2017).

Inhibition of ERK while simultaneously adding IGF1 resulted in increased length of metatarsals, especially of the cartilage area as compared to the untreated control. In addition, we observe at least the same levels of matrix production as in control cells, in some metatarsals even higher. We have not quantified the number of cells, as there are many cells in these metatarsals at this developmental stage, but by eye it seems that there are more cells in the metatarsals treated with PD98059+IGF, which is reflected in the zone measurements that shows that the length of all zones is increased in these metatarsals.

For treatments with BMP7 + PTHrP (treatment 1), ECHO predicted that in the RUNX2+ state there is little to no receptor for PTHrP (called “PPR” in ECHO): node activity of “PPR Prot” is very low, about 11/100. So, because there are few receptors available, just adding PTHrP to ECHO is not enough to activate the PTHrP pathway (PPR activity stays at 0 even with 100% activity of PTHrP). And indeed, we saw this already in Figure 4: the PTHrP pathway is “strong enough” to change a RUNX2+ state into SOX9+ by itself, but we can activate it only by activating PPR (PTHrP Receptor) directly. We also noted that BMP is already fully active in the RUNX2+ state, but it is of note that the BMP we have in ECHO represents both BMP2 and 7, with a bias toward BMP2 effects as those are more often described in literature.

PTHrP is a well-known regulator of cartilage development and it is shown to inhibit the differentiation of proliferating chondrocytes into pre-hypertrophic chondrocytes (Lanske et al., 1996; Weir et al., 1996; Mackie et al., 2008; Welting et al., 2018). The effect of PTHrP in regulating proliferation of preventing hypertrophy is dependent on the dose (Loveys et al., 1993). As compared to what others use, we used a very high concentration of PTHrP (1 μM). At this concentration, we expected to see an increase in proliferation, but less so in prevention of hypertrophy (Loveys et al., 1993).

Seeing the current interest in BMP7 as a possible treatment for OA (Caron et al., 2013; Huang et al., 2018; Caron et al., 2021) and its role in cartilage development (Kronenberg, 2003; Mackie et al., 2008; Kozhemyakina et al., 2015; Welting et al., 2018), we were especially curious to see the combined effect of BMP7 and PTHrP on cartilage development of the metatarsal bone. Although we observed an increase in growth as compared to our untreated controls, histological staining showed a slight but unsignificant decrease in proliferative zone and an increase in the size of the hypertrophic zone as compared to our control. Interestingly, we observed a significant decrease in matrix production as compared to the controls albeit not as low as for the DLX5+/PKA-treatment.

Even though predicted by ECHO, this result was slightly surprising as we had expected that both the use of BMP7 as well as PTHrP would induce chondrocyte proliferation and prevent hypertrophy, which has been shown before in embryonic mouse metatarsals (Loveys et al., 1993; Haaijman et al., 1999). This seemingly discrepant data can at least be partially explained by the differences in the experiments. While Haaijman et al. treated mouse embryonic (E15) metatarsals, at which stage no endochondral ossification centre is present, with 40 ng/ml BMP7 and showed that this was independent on the expression of PTHrP, we treated rat new-born metatarsals, which at that point already contain the secondary ossification centre, with a 2.5 fold higher dose of BMP7. At this developmental stage, the effect may not be as severe as at earlier developmental stages, since terminal differentiation has already occurred for at least a subset of cells. Although we do not observe a relatively large proliferative zone, we did observe the largest overall growth of the metatarsals for this PTHrP +BMP7 treatment, most notably in the cartilage zone, indicating that BMP7+PTHrP indeed stimulated proliferation rather than cartilage matrix production. To see the effect of these treatments on stem cell fate choices, these experiments should be performed in (mesenchymal or iPSC) stem cells, which was not possible due to lab closures in this COVID-19 dominated year.

## Conclusion

In this work, we describe ECHO as an executable model to explore network dynamics, derive hypotheses, design experiments, and predict the outcomes of these experiments. Our manuscript shows that building activity-based signaling networks of a cell provides important information the role of signals in cell fate decisions. Moreover, *in silico* experiments allow researchers to test many hypotheses before validating them in the wet-lab, thereby reducing time and costs for experiments. We used model-checking to prioritize combinatorial treatments that were shown to induce a switch between transient and permanent cartilage. We validated the model predictions that treatment with IGF1, while inhibiting ERK1/2 has a positive effect on cartilage formation and growth, with a relative decrease in hypertrophy as compared to control samples, while activation of DLX5 while inhibiting PKA results in impaired growth, increased cartilage hypertrophy and prevented cartilage matrix formation. Interestingly, ECHO predicted the combination of PTHrP +BMP7 was not sufficient to switch from a RUNX2+ to a SOX9+ state, even though we intuitively expected that this combination of treatment would strongly enhance cartilage formation. This shows that computational modelling can not only be used for finding new mechanisms, but also for taking away human bias by providing objective model predictions.

## Data Availability

The original contributions presented in the study are included in the article/Supplementary Material, further inquiries can be directed to the corresponding author.
