# Supplementary figures and images for: An ECHO of Cartilage: In Silico Prediction of Combinatorial Treatments to Switch Between Transient and Permanent Cartilage Phenotypes With Ex Vivo Validation
Source: Front Bioeng Biotechnol. 2021 Nov 15;9:732917. doi: 10.3389/fbioe.2021.732917 (PMC8634894; doi:10.3389/fbioe.2021.732917)

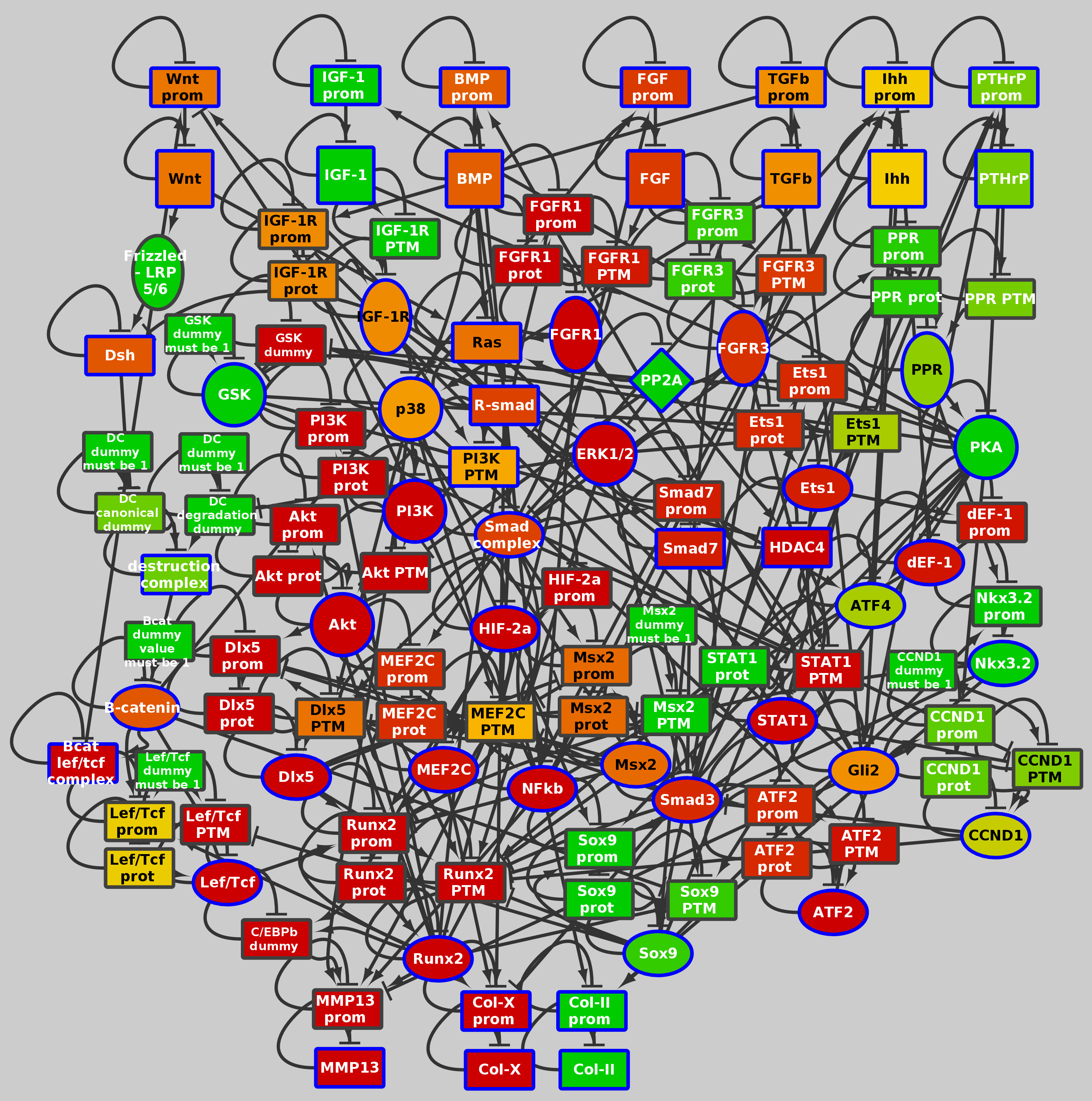

Supplement: Supplementary file 2 [file Image3.JPEG]

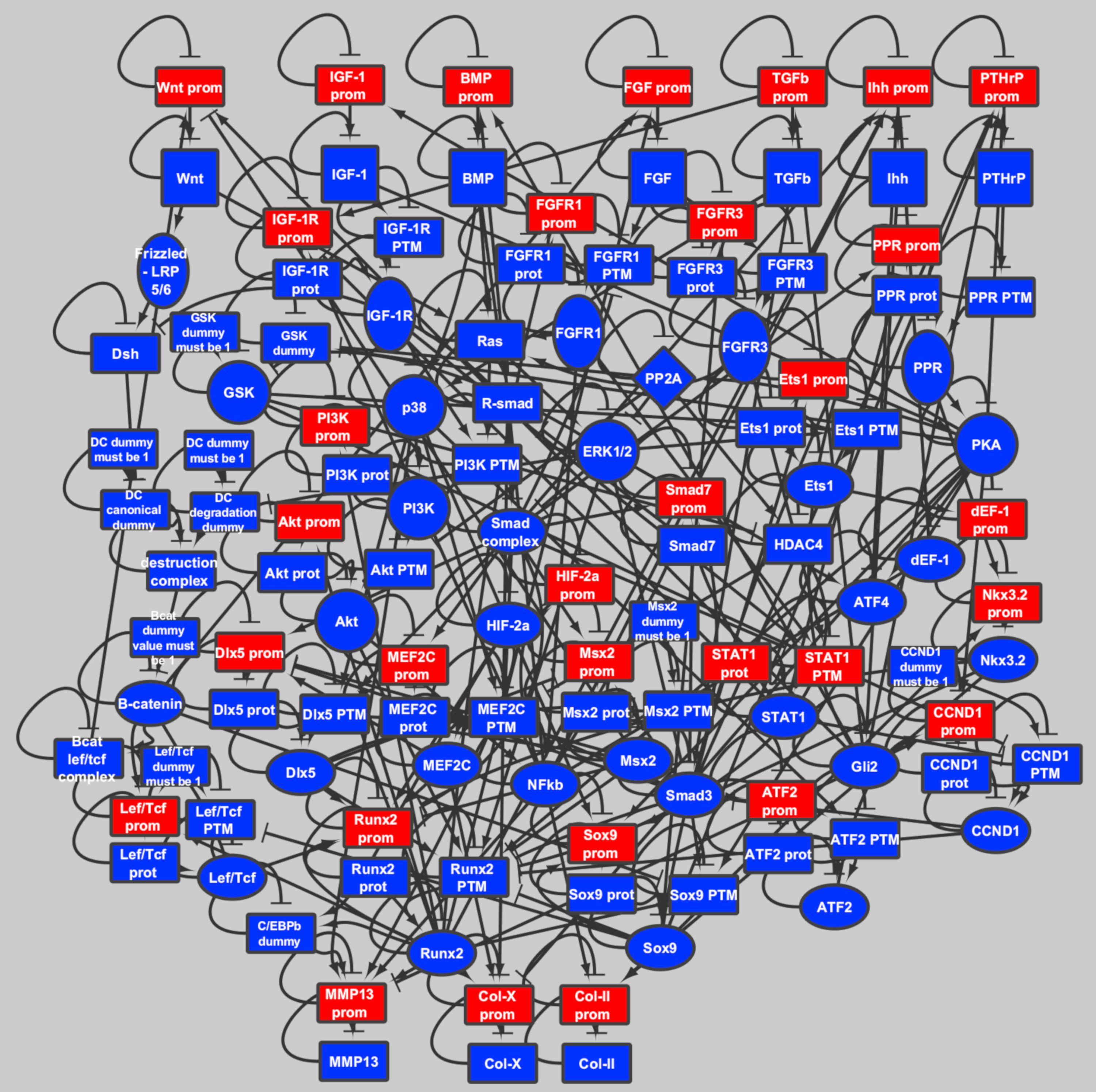

Supplement: Supplementary file 7 [file Image4.JPEG]

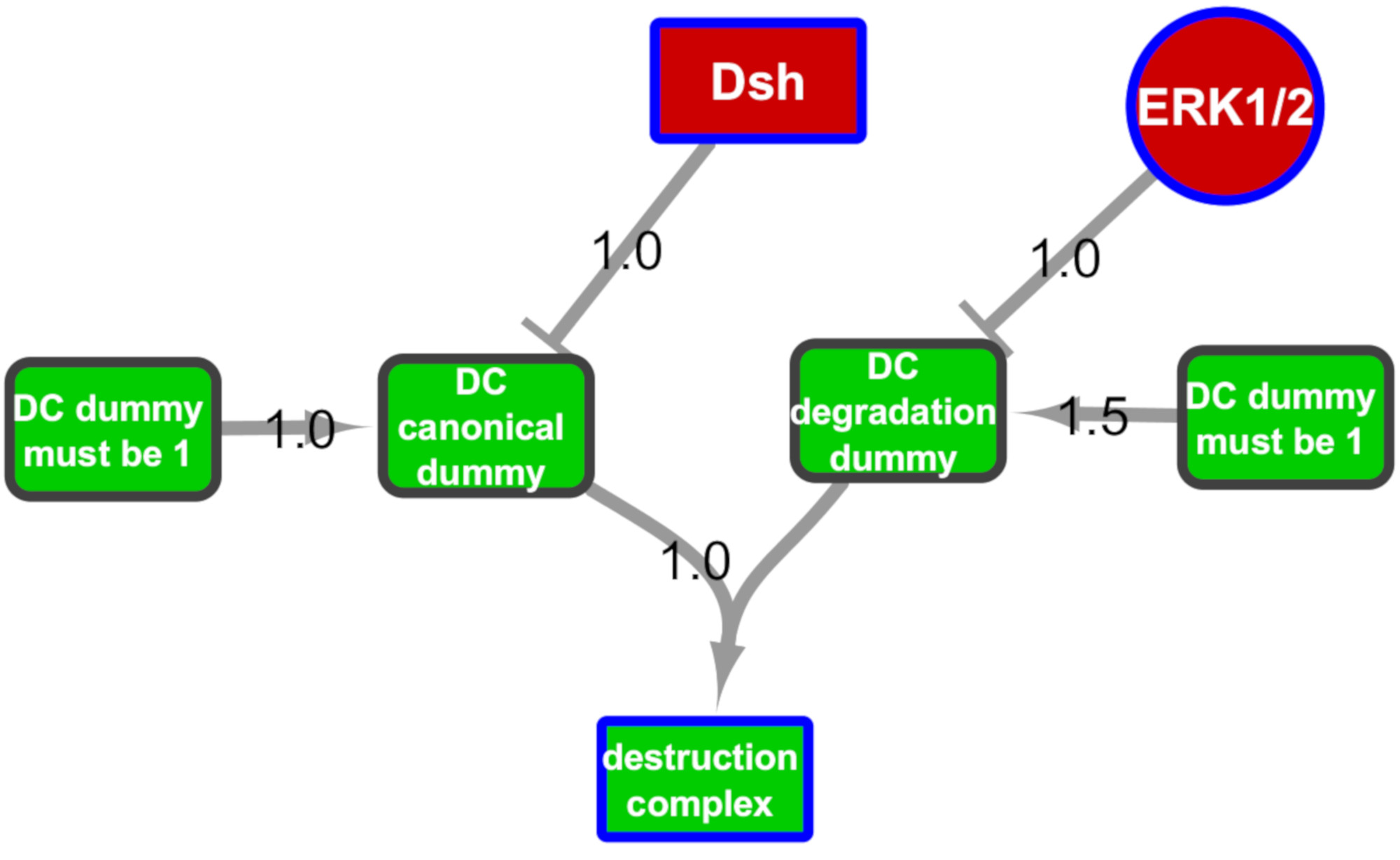

Supplement: Supplementary file 9 [file Image2.JPEG]

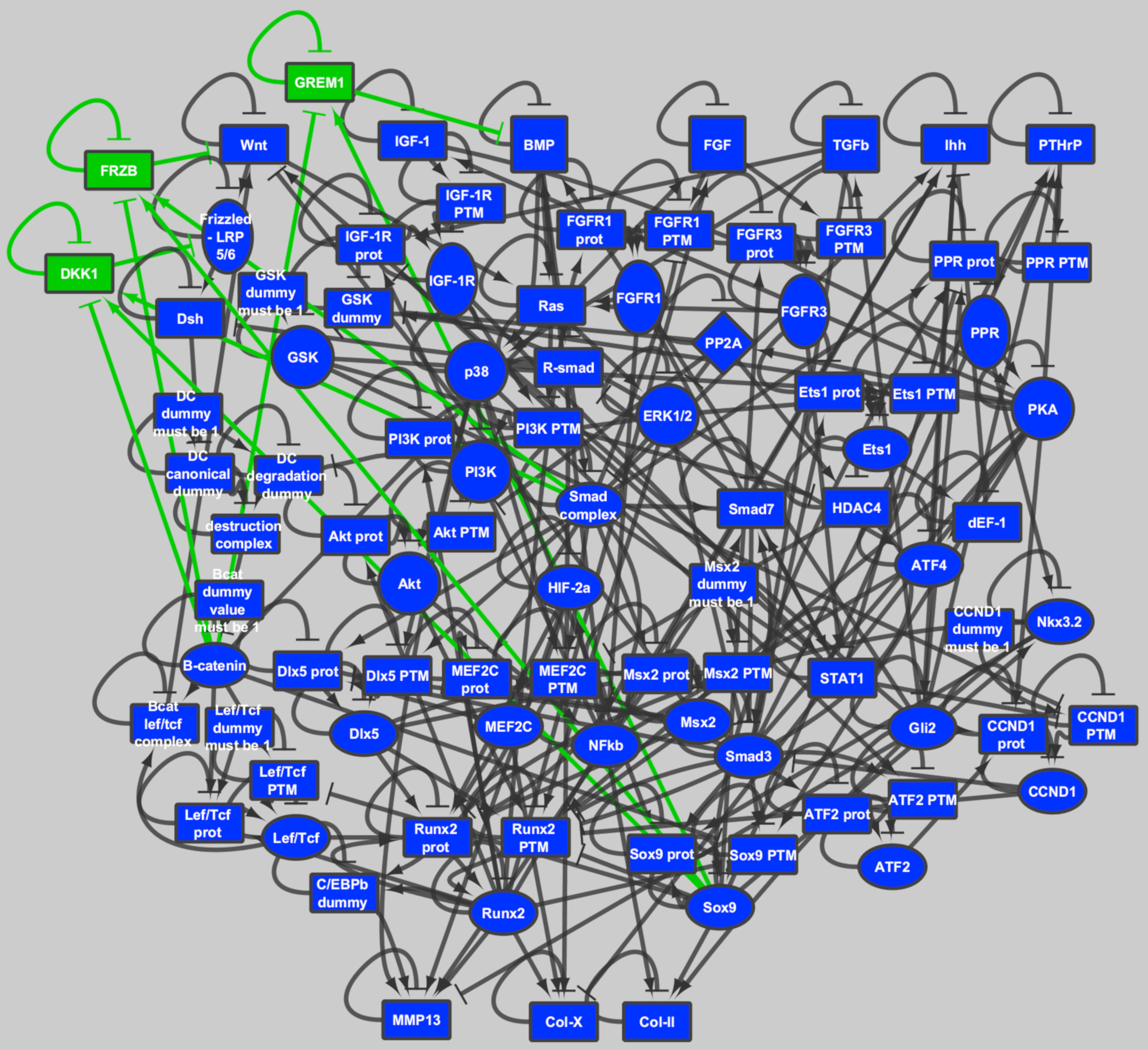

Supplement: Supplementary file 10 [file Image5.JPEG]

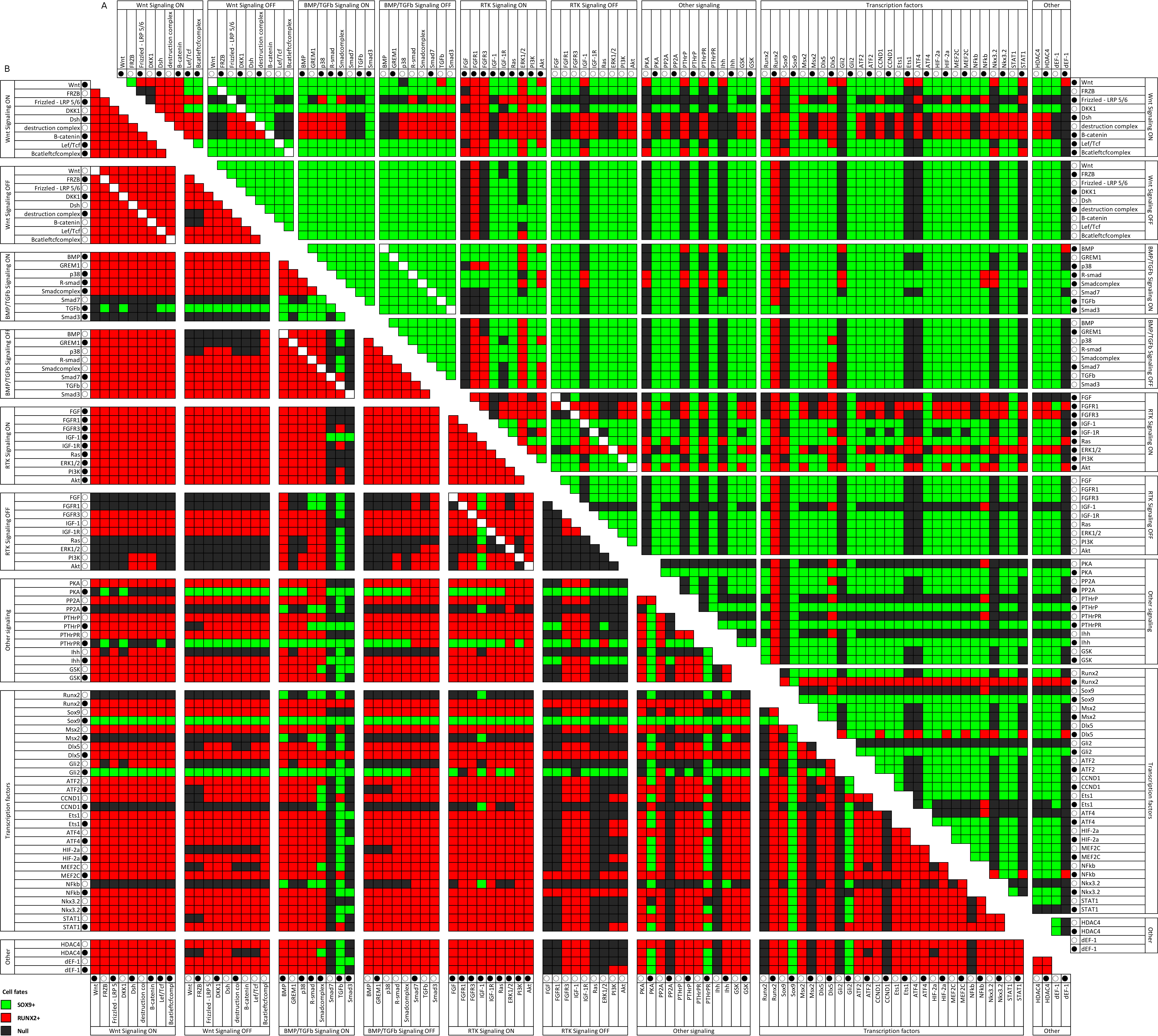

Supplement: Supplementary file 13 [file Image6.PNG]
