# Supplementary material for: An ECHO of Cartilage: In Silico Prediction of Combinatorial Treatments to Switch Between Transient and Permanent Cartilage Phenotypes With Ex Vivo Validation
Source: Front Bioeng Biotechnol. 2021 Nov 15;9:732917. doi: 10.3389/fbioe.2021.732917 (PMC8634894; doi:10.3389/fbioe.2021.732917)
Supplement: Supplementary file 12 [file DataSheet2.docx]

Supplementary Material

# Supplementary Figures and Tables

**Supplementary figures 1-6**

**Supplementary Table 1:** parameters of nodes of initial ECHO model after conversion from the Boolean network described in (Kerkhofs et al., 2012).

**Supplementary Table 2:** parameters of edges of initial ECHO model after conversion from the Boolean network described in (Kerkhofs et al., 2012).

**Supplementary Table 3:** parameters nodes and edges of the GP model (model 1)

**Supplementary Table 4:** parameters of edges of the AC model (model 4).

**Supplemental materials: models and scripts van model checking**

## Supplementary Figures

**Supplementary Figure 1.** ANIMO workflow. When the “Analyze network” button is clicked in the Cytoscape-based interface, ANIMO translates the network into a Timed Automata model, then uses UPPAAL to analyze it. The numerical results are parsed, and used to produce a graph that shows the activity levels of selected network nodes over the simulation period. A slider under the graph can be moved to highlight particular points in the simulation (vertical red line): node colors in the network adapt according to their activity at that time-point.

**Supplementary Figure 2.** Nodes and edges used in ECHO to represent the activity of the Destruction complex following the original formula (1-Dsh)*Min((1.5 - ERK), 1). Activations are represented by 🡪, and inhibitions by ⊣. The k-values are written on the edges. The double-edge entering "destruction complex" represents an AND gate: both “DC canonical dummy” and “DC degradation dummy” need to be active for “destruction complex” to become active.

**Supplementary Figure S3.** The first version of the Executable CHOndrocyte, ECHO, containing 120 nodes and 343 interactions.

**Supplementary Figure S4.** Model 1 was obtained by removing the nodes representing expression or post-translational modification processes that are not modelled in ECHO (in red). In particular, the “prom” nodes (which represented promotors on the DNA) have been removed, and the influences on protein production have been redirected to the “prot” nodes instead. Please refer to the files in the supplemental data folder to open the model in ANIMO. The initial activities of the nodes and parameters of the edges can be found in supplementary tables 1 and 2 (separate Excel files).

**Supplementary Figure 5.** Model 2 was obtained by addition of the WNT antagonists DKK1 and FRZB, and the BMP antagonist GREM1 (shown in green).

**Supplementary Figure 6.** Combination of perturbations that cause A. a SOX9+ state to switch to a RUNX2+ state or B. a RUNX2+ state to switch to a SOX9+ state in model 4. Each pair of nodes in the network was perturbed in all combinations of knock-out (empty circle, ○) and constitutive activation (full circle, ●), while all other nodes were initialized as in the SOX9 state (A) or Runx2+ state (B). After one simulation, the resulting stable state was recorded.

**
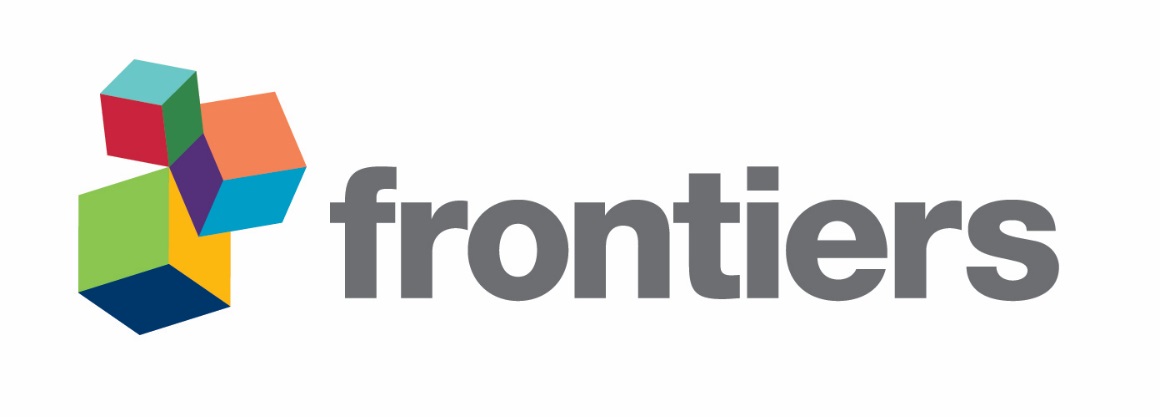
**
